# Silencing of *OsGRXS17* in rice improves drought stress tolerance by modulating ROS accumulation and stomatal closure

**DOI:** 10.1038/s41598-017-16230-7

**Published:** 2017-11-21

**Authors:** Ying Hu, Qingyu Wu, Zhao Peng, Stuart A. Sprague, Wei Wang, Jungeun Park, Eduard Akhunov, Krishna S. V. Jagadish, Paul A. Nakata, Ninghui Cheng, Kendal D. Hirschi, Frank F. White, Sunghun Park

**Affiliations:** 1 Department of Horticulture and Natural Resources, Kansas State University, Manhattan, KS 66506 USA; 2 Department of Plant Pathology, Kansas State University, Manhattan, KS 66506 USA; 3 Department of Agronomy, Kansas State University, Manhattan, KS 66506 USA; 4 United States Department of Agriculture/Agricultural Research Service, Children’s Nutrition Research Center, Department of Pediatrics, Baylor College of Medicine, Houston, TX 77030 USA; 5 Present Address: Department of Plant Pathology, Kansas State University, Manhattan, KS 66506 USA; 6 Present Address: Cold Spring Harbor Laboratory, Cold Spring Harbor, NY 11724 USA; 7 Present Address: Department of Plant Pathology, University of Florida, Gainesville, FL 32611 USA

## Abstract

Glutaredoxins (GRXs) modulate redox-dependent signaling pathways and have emerged as key mediators in plant responses to environmental stimuli. Here we report that RNAi-mediated suppression of *Oryza sativa GRXS17* (*OsGRXS17*) improved drought tolerance in rice. Gene expression studies showed that *OsGRXS17* was present throughout the plant and that transcript abundance increased in response to drought stress and abscisic acid (ABA) treatment. Localization studies, utilizing GFP-OsGRXS17 fusion proteins, indicated that OsGRXS17 resides in both the cytoplasm and the nuclear envelope. Under drought stress conditions, rice plants with reduced *OsGRXS17* expression showed lower rates of water loss and stomatal conductance, higher relative water content, and enhanced survival compared to wild-type controls. Further characterization of the *OsGRXS17* down-regulated plants revealed an elevation in H_2_O_2_ production within the guard cells, increased sensitivity to ABA, and a reduction in stomatal apertures. The findings demonstrate a critical link between OsGRXS17, the modulation of guard cell H_2_O_2_ concentrations, and stomatal closure, expanding our understanding of the mechanisms governing plant responses to drought.

## Introduction

Drought is a critical limiting factor for food production. In rice, for example, drought is estimated to affect 23 million hectares and, consequently, threatening the food security of 3 billion people^1,2^. Plants have evolved multiple strategies to adapt to drought stress, such as maintaining water potential through deep root systems and reducing water loss by promoting stomata closure in the aerial portions of the plant^3^. Stomatal closure is one of the most important protection mechanisms that plants utilize to minimize water loss. The pathway of stomatal closure is mediated by reactive oxygen species (ROS) and abscisic acid (ABA) signaling^4–7^. Upon drought stress, ABA levels in the plant increase due to increased biosynthesis and decreased degradation^8,9^. The accumulated ABA is perceived by the ABA receptors RCAR, PYR1, and PYL, which, in turn, interact with a group of type 2C protein phosphatases (PP2C) to relieve PP2C-mediated inhibition of the SNF1-related protein kinase OPEN STOMATA1 (OST1)/SnRK2^10,11^. Activated OST1 targets the plasma membrane-bound NADPH oxidase (RBOH), which catalyzes H_2_O_2_ production^12^. H_2_O_2_-activated calcium channels increase the calcium level in the cytosol of guard cells, resulting in membrane depolarization, activation of K^+^_out_ channels and the efflux of organic acids, and, ultimately, trigger stomatal closure^13^. Although ROS serve as important signaling molecules in the control of stomatal aperture status as well as other stress-related responses^14–16^, uncontrolled or prolong exposure to ROS can cause oxidative damage to lipids, proteins, and DNA^17,18^.

To mitigate cellular damage, plants have evolved a versatile ROS scavenging system that functions in conjunction with the mechanisms regulating ROS production to control cellular ROS concentrations^14–16^. Glutaredoxins (GRXs) are ubiquitous oxidoreductases in the thioredoxin (TRX) family and are involved in maintaining cellular redox homeostasis and regulating the redox-dependent signal pathway. GRXs utilize the reducing power of glutathione (GSH) to catalyze reversible reduction of disulfide bonds of the cognate target proteins^19–21^. GRXs also act as redox regulators in different aspects of plant growth, such as iron homeostasis, heavy metal detoxification, plant development, and plant-pathogen interaction^19,21,22^. Genomic sequence data have revealed families of GRX genes in higher plants, including *Arabidopsis thaliana* with fifty predicted GRX genes, *Populus trichocarpa* with thirty-six, and *Oryza sativa* with twenty-seven^23–25^. Based on the predicted active sites, plant GRXs can be subdivided into four groups^26,27^. GRXs of class I and class II have so-called CxxC/S and CGFS active sites, respectively, and are conserved in all photosynthetic organisms. Class III GRXs are specific to higher plants and have a peculiar CCxx active site. GRXs of class IV harbor a CxDC/S active site. Many studies have demonstrated Class II GRXs involvement in stress adaptation of plants^28–32^.

GRXS17 is a Class II GRX that plays an essential role in chilling stress, heat stress, and photoperiod responses^33–36^. However, a function for GRXS17 in drought stress response has not been investigated. In this study, we examined the spatial expression of *OsGRXS17* in rice plants under normal and drought stress conditions and used *OsGRXS17* silenced rice plants to investigate a role for OsGRXS17 in drought stress responses.

## Results

### *OsGRXS17* is a Functional Homolog of *AtGRXS17*

**Figure 1:**
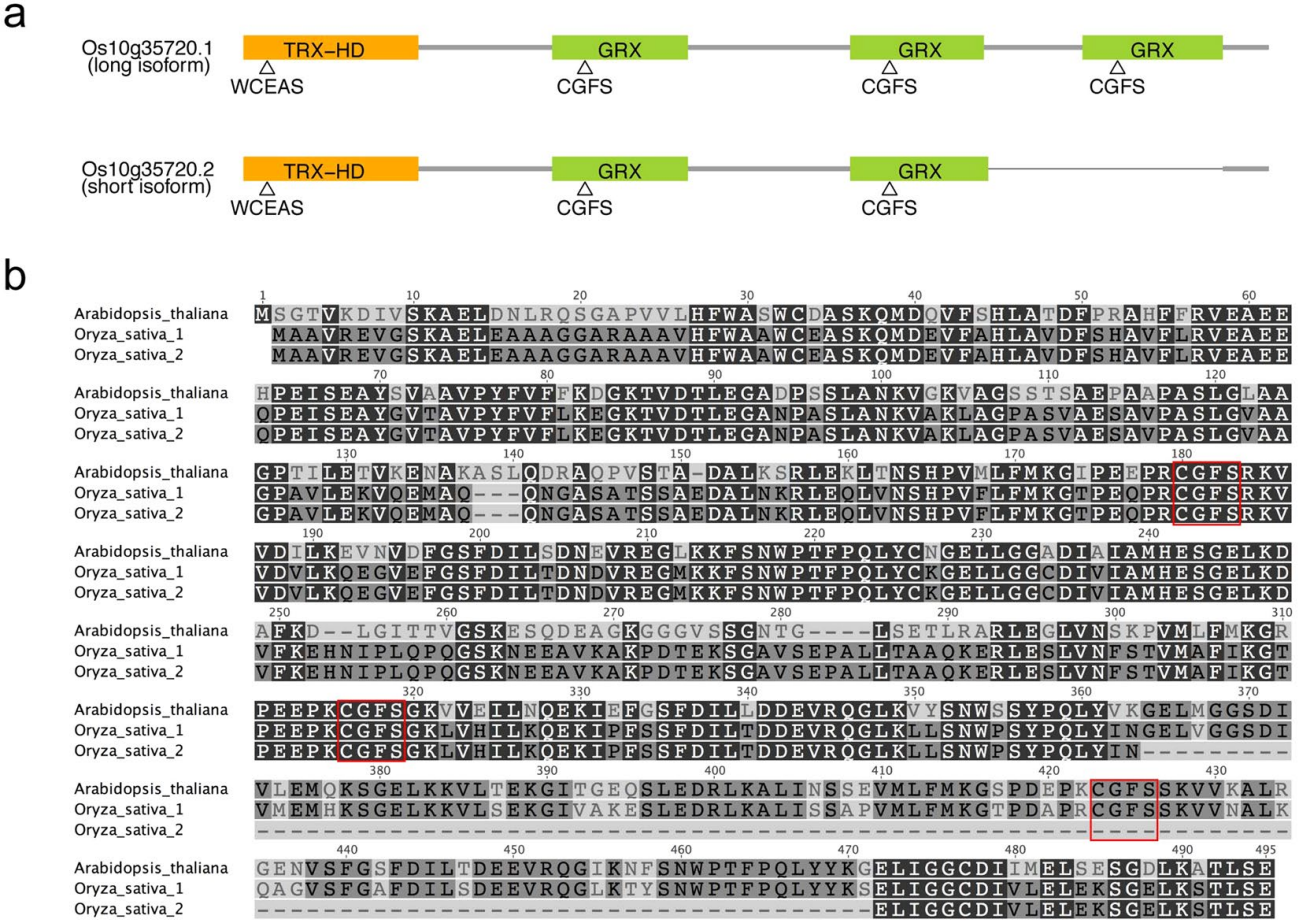
Domain structure of two isoforms of OsGRXS17 and amino acid sequence alignment of AtGRXS17 and two isoforms of OsGRXS17. (**a**) TRX-HD represents the TRX-like homology domain, GRX represents the monothiol-GRX domain, and triangle indicates the position of active sites. (**b**) Completely conserved residues are indicated by black boxes and residues conserved in the majority of sequences are indicated by gray boxes. The CGFS active domains are indicated by red border rectangles.

**Figure 2:**
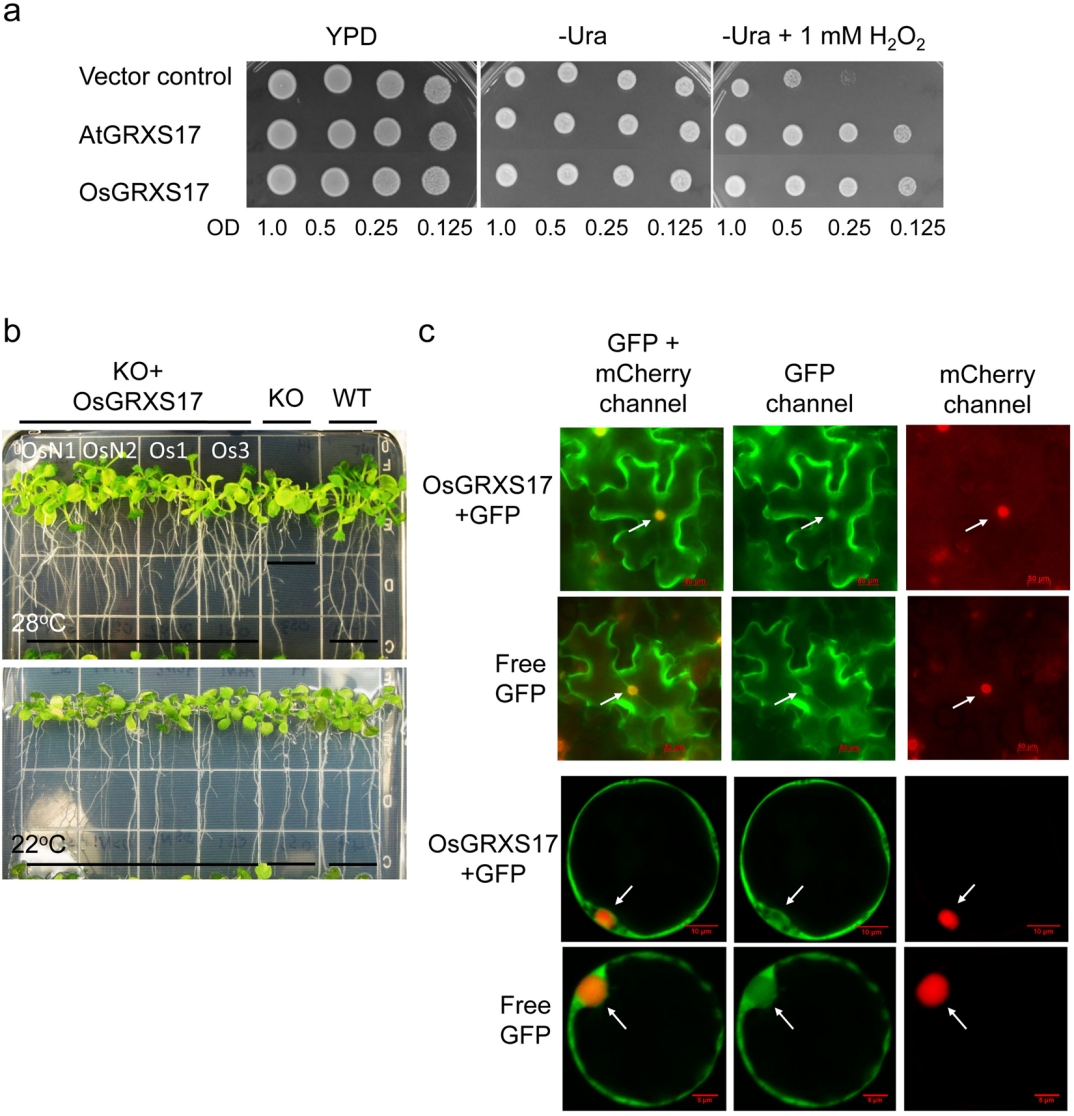
OsGRXS17 rescues the hypersensitivity of yeast *grx3grx4* mutant and defective phenotypes of *Arabidopsis atgrxs17* mutant. (**a**) Vector control-, *AtGRXS17*- and *OsGRXS17*-expressing *grx3/grx4* cells were grown on YPD, SC-Ura or SC-Ura +1 mM H_2_O_2_ media for 3 days at 30 °C. OsGRXS17 was able to suppress the sensitivity of *grx3grx4* cells to oxidative stress. AtGRXS17 was used as a positive control here. (**b**) Rice *OsGRXS17* suppressed *atgrxs17* KO mutant phenotypes grown under elevated temperature (28 °C), while *atgrxs17* KO seedlings displayed short primary roots and the growth of seedlings was inhibited when grown under 28 °C. (**c**) Transient expression of GFP-OsGRXS17 and free GFP in tobacco epidermal cells and rice protoplast. Scale bars = 50 µm. As expected the free GFP control was localized in both the cytoplasm and the nuclei. A vector harboring 35S::mCherry::NLS (the mCherry red fluorescent protein linked to a nuclear localization signal) was used as a control for nuclear localization in transient co-expression assays (Fig. 2c, right). The arrows highlight the nuclei.

Examination of the Rice Functional Genomic Express Database (http://signal.salk.edu/cgi-bin/RiceGE) revealed a rice homolog, *OsGRXS17* (Os10g35720), of the *Arabidopsis AtGRXS17* with 67.8% identity at the amino acid level (Fig. S1). OsGRXS17 (Os10g35720.1, long isoform) consists of 491 amino acid residues with an N-terminal TRX-like homology domain (HD) and three tandem GRX-HDs (Fig. 1a,b). Similar homologs were detected in maize, tomato, and potato (Fig. S1). Evidence was found in rice for an alternatively spliced short isoform (Os10g35720.2), which encodes a protein of 384 amino acids and consists of the TRX-like HD and only two monothiol GRX-HDs (Fig. 1a,b). In yeast, *grx3grx4* double mutants, when exposed to H_2_O_2_, show markedly reduced cell growth, which can be recovered by expression of the *Arabidopsis AtGRXS17* ^34^. The long isoform of *OsGRXS17* showed similar properties by restoring growth in the presence of H_2_O_2_ when expressed in the double-mutant strain (Fig. 2a). *Arabidopsis* T-DNA insertion mutant (*atgrxs17*) lines are defective in vegetative growth and development and sensitive to elevated temperature stress^35^. OsGRXS17 complements the loss of *AtGRXS17* in the *atgrxs17* line, rescuing defective mutant phenotypes under heat stress (Fig. 2b). When expressed as a GFP fusion protein GFP-OsGRXS17 is detected in both the cytoplasm and nuclei of tobacco leaf epidermal cells and rice protoplasts (Fig. 2c), similar to the results that were seen with AtGRXS17^33,34^.

### Expression Pattern of *OsGRXS17* in Response to Stress and Plant Hormones

**Figure 3:**
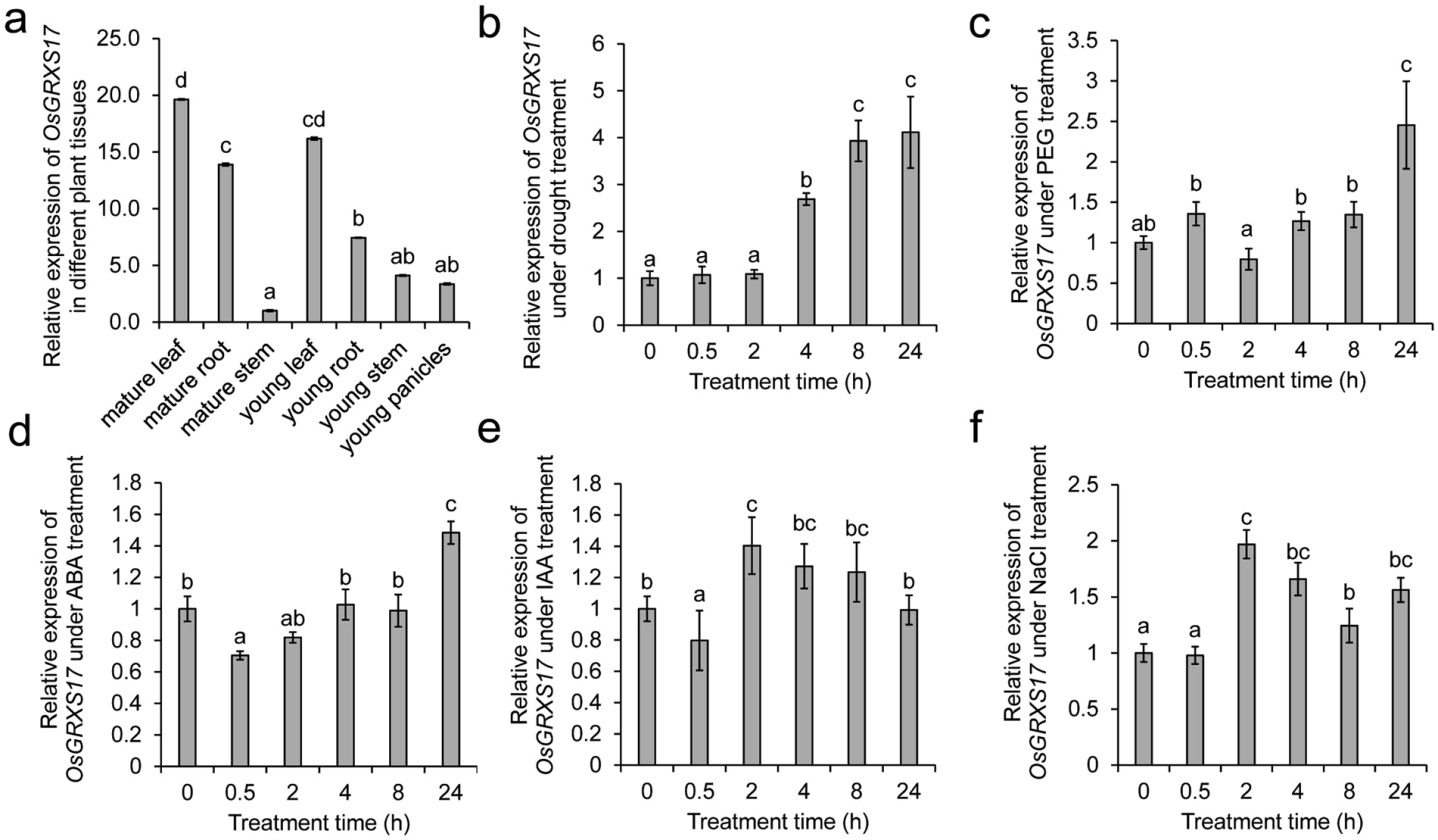
Expression pattern of *OsGRXS17* in different tissues and under different stress treatments. (**a**) Relative expression level of *OsGRXS17* in different tissues of two-week-old wild-type rice plants by qRT-PCR analysis. Relative expression level of *OsGRXS17* in the shoots of two-week-old wild-type rice plants treated with drought stress (**b**), PEG (**c**), ABA (**d**), IAA (**e**) and NaCl (**f**). Data are expressed as relative values based on wild-type plants before treatments as reference sample set to 1.0. Error bars represent the means ± SD (*n* = 3). Values with the same lowercase letter are not significantly different at the p-value < 0.05 with Tukey’s test.

Although the gene was annotated to have two alternative splicing variants, we were only able to detect the long isoform in the shoots of the 14-day-old seedlings that we studied. Thus, all the quantitative RT-PCR (qRT-PCR) data presented here are about the long isoform. The expression pattern of the *OsGRXS17* gene, as determined by qRT-PCR, indicated that leaves and roots had higher expression levels than either stems or young panicles (Fig. 3a). The expression pattern of *OsGRXS17* in rice shoots was also examined under reduced watering (Fig. 3b), in the presence of polyethylene glycol (PEG) (Fig. 3c), after application of ABA (Fig. 3d), indole-3-acetic acid (IAA) (Fig. 3e), and high salinity treatments (Fig. 3f). *OsGRXS17* transcript levels were elevated 2 h after the start of drought stress treatment and continued to increase until 8 h, at which time the transcript levels plateaued (Fig. 3b). PEG or ABA treatments led to high levels of expression of *OsGRXS17* after 24 h (Fig. 3c,d). *OsGRXS17* levels transiently increased 2 h after treatments with IAA or salt (Fig. 3e,f).

### Generation of *OsGRXS17* Silenced RNAi Rice Plants

**Figure 4:**
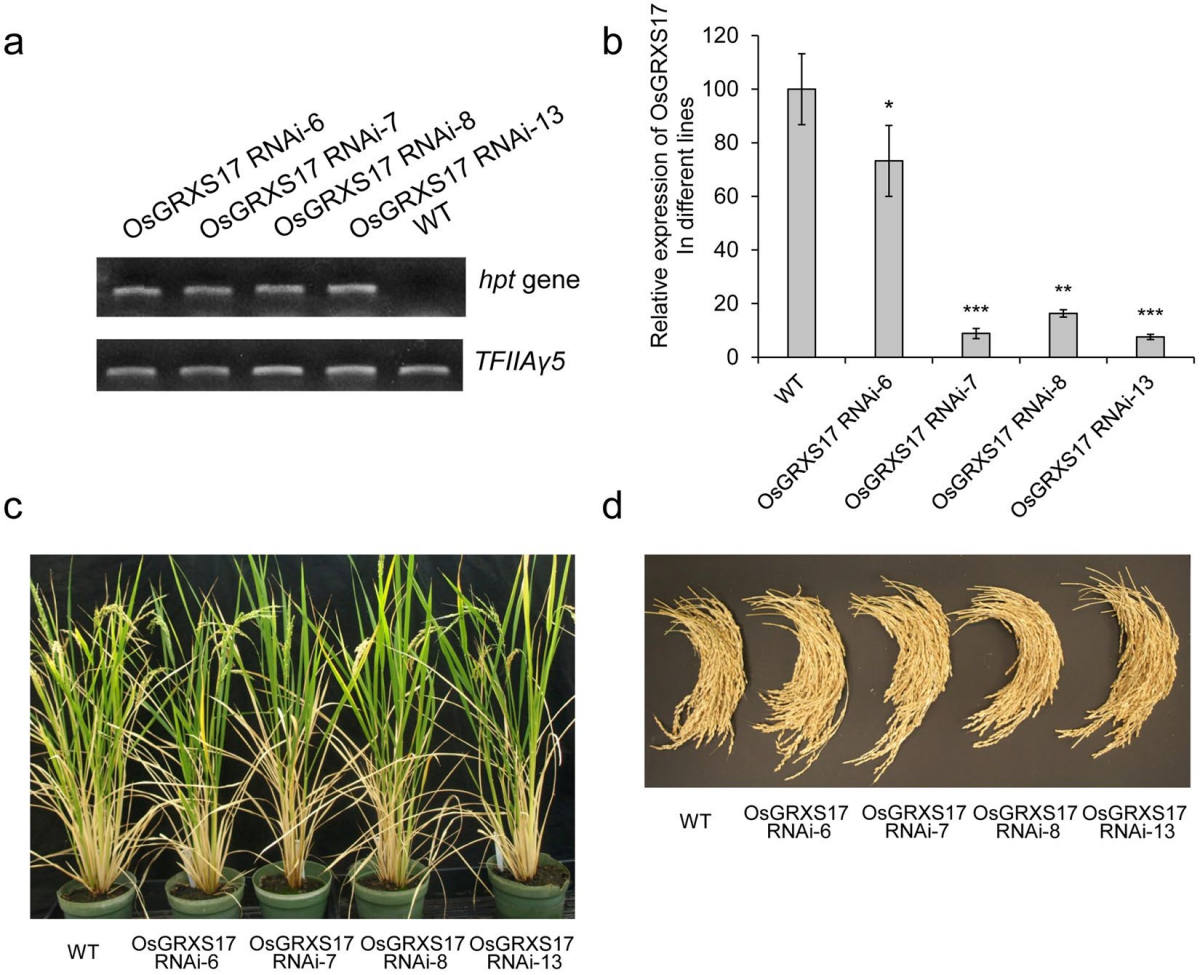
Expression levels of *OsGRXS17* and the phenotypes of *OsGRXS17* silenced and wild-type rice lines under normal growth conditions. (**a**) The expression of a *hygromycin phosphotransferase* (*hpt*) gene was confirmed by RT-PCR in wild-type and four selected *OsGRXS17*-silenced rice plants (full-length gels are presented in Supplementary Figure 8). The *TFIIAγ5* gene was included as control for uniform RT-PCR conditions (bottom). (**b**) The relative expression level of *OsGRXS17* was measured by qRT-PCR in wild-type and four *OsGRXS17* silenced rice plants. (**c**) The phenotype of wild-type and *OsGRXS17*-silenced rice plants are indistinguishable at reproductive stage. (**d**) Total panicles were collected from three plants per line and *OsGRXS17* silenced does not affect the yield. Error bars represent the means ± SD (*n* = 3). Asterisks (*, **, ***) represent statistically significant differences between wild-type and *OsGRXS17* silenced lines (Student’s *t-test*, *P < 0.05, **P < 0.01, ***P < 0.001).

Expression of *OsGRXS17* was altered by expression of inhibitory short hairpin RNAs derived from *OsGRXS17* gene under the control of the maize ubiquitin (*Ubi*) promoter. The construct was transformed into *Oryza sativa* L. *Japonica* cv. Nipponbare, self-pollinated, and the progeny lines were genotyped for the presence of T-DNA (Fig. 4a). The copy number of T-DNA insertions of those transgenic lines was determined by Southern blot analysis using a hygromycin (*hpt*) gene-specific probe (Fig. S2). Lines *OsGRXS17* RNAi-6 and *OsGRXS17* RNAi-7 contain a single transgene insertion, while *OsGRXS17* RNAi-8 and *OsGRXS17* RNAi-13 had multiple integration events (Fig. S2). Expression levels of *OsGRXS17* in lines *OsGRXS17* RNAi-6, -7, -8 and -13 fell into two groups as measured by qRT-PCR (Fig. 4b). Lines *OsGRXS17* RNAi-7, -8 and -13 had reduced levels of *OsGRXS17* expression at ~80–90% compared to the wild-type plants, while *OsGRXS17* RNAi-6 had reduced levels of *OsGRXS17* expression at ~25% compared to the wild-type plants. To examine if the expression of other endogenous *Oryza sativa GRX* genes, which are the most closely related to *OsGRXS17*, is altered in the *OsGRXS17* RNAi lines, the expression levels of four endogenous *OsGRX* genes (subgroup II *OsGRXS14*, *OsGRXS15.1*, *OsGRXS15.2*, and *OsGRXS16*) identified by phylogenetic analysis (Fig. S3) and amino acid sequence alignment (Fig. S4a) were measured by qRT-PCR. No significant differences in the expression level were found between wild-type and *OsGRXS17* RNAi rice plants (Fig. S4b). The phenotypes of the *OsGRXS17* silenced plants were indistinguishable from wild-type plants at both vegetative and reproductive stages under normal growth conditions (Fig. 4c,d). The agronomic traits analyses, including panicle number per plant, main panicle length, grain number of main panicle, seed set percentage of main panicle, 100-grain weight of main panicle and panicle weight per plant, indicated no differences between *OsGRXS17* silenced and wild-type rice plants (Fig. S5).

### Silenced Expression of *OsGRXS17* Enhances Drought Stress Tolerance in Rice

**Figure 5:**
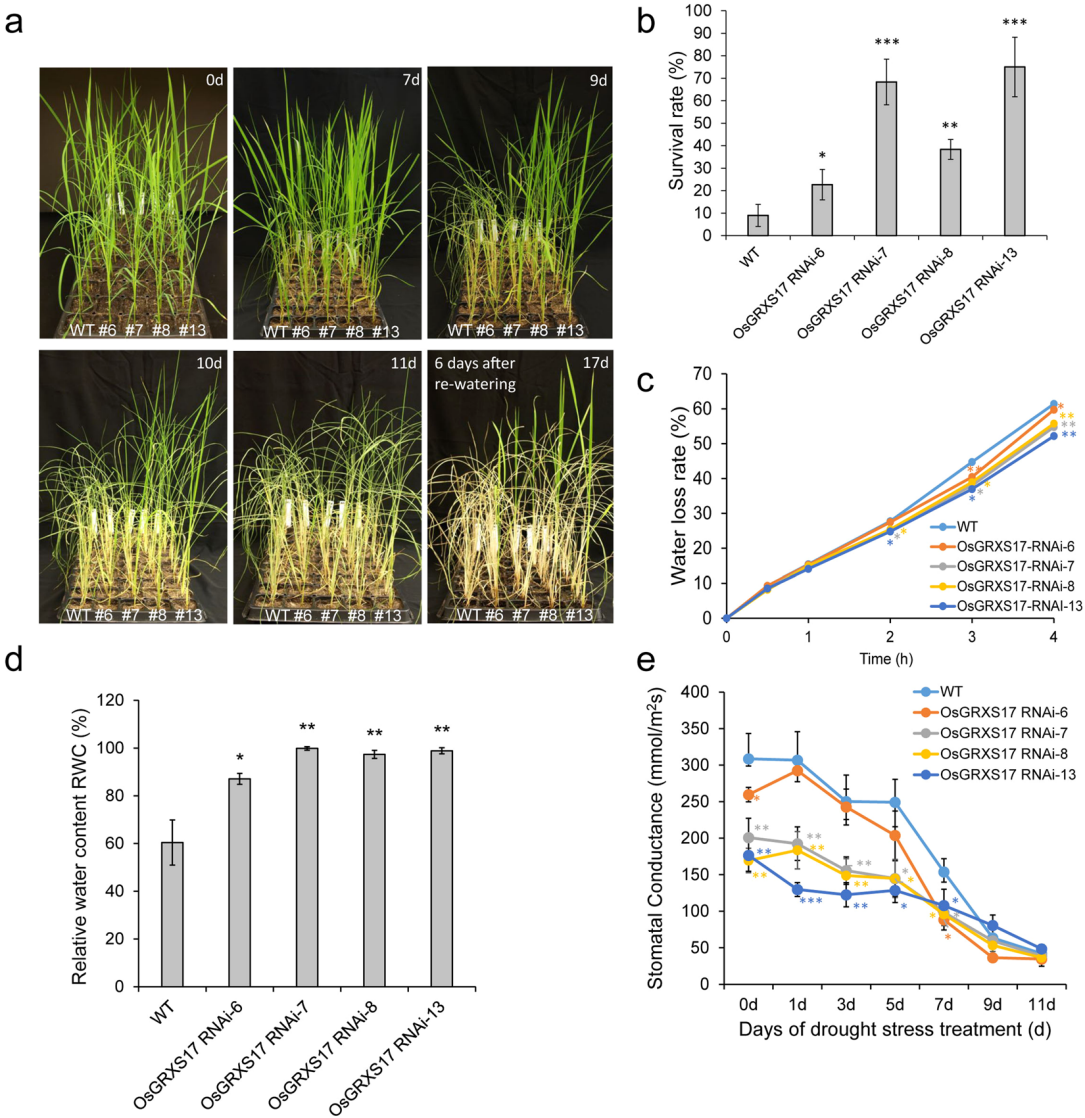
The *OsGRXS17* silenced rice plants show tolerance to drought stress. (**a**) Drought stress treatment of wild-type and *OsGRXS17* silenced rice plants. The four-week-old plants (0d) were treated by withholding water for 11 days and re-watered for 6 days to recover. (**b**) The survival rate of the wild-type and *OsGRXS17* silenced rice plants after 11 days of drought stress treatment and 6 days of re-watering. (**c**) Water loss rate in the leaves cut from four-week-old wild-type and *OsGRXS17* silenced rice plants (*n* = 3 repeats). (**d**) The relative water content of four-week-old wild-type and *OsGRXS17* silenced rice plants after 5 days of drought stress treatment (*n* = 3 repeats). (**e**) Stomatal conductance of four-week-old wild-type and *OsGRXS17* silenced rice plants under drought stress for 11 days (*n* = 3 repeats). Error bars represent the means ± SD (*n* = 3). Asterisks (*, **, ***) represent statistically significant differences between wild-type and *OsGRXS17* silenced lines (Student’s *t-test*, *P < 0.05, **P < 0.01, ***P < 0.001).

T2 homozygous plants of the four *OsGRXS17* silenced lines and wild-type plants were subjected to drought stress treatment. The lines were visually indistinguishable before withholding water (Fig. 5a, 0 d). After withholding water for 9 days, leaves of *OsGRXS17* RNAi-7, -8 and -13 rice plants remained green and turgid, while wild-type and *OsGRXS17* RNAi-6 rice plants wilted (Fig. 5a, 9 and 10 d). At 11 days, all plants displayed wilting (Fig. 5a, 11 d). Wild-type and *OsGRXS17* silenced rice plants were re-watered after 11 days to test recovery from drought stress treatment (Fig. 5a, 17 d with 6 days of re-watering). Six days after the new watering regimen, the percentage of plants that visibly recovered on the basis of new green leaf production was measured (Fig. 5a, 17 d with 6 days of re-watering and Fig. 5b). Silenced plants showed recovery (survival) rates in a range of 23–75%, while 8% of the wild-type plants survived (Fig. 5b). Further, lines *OsGRXS17* RNAi-7, -8 and -13 had higher survival rates than *OsGRXS17* RNAi-6. To evaluate the physiological responses associated with drought tolerance in *OsGRXS17* silenced plants, water loss in leaves of wild-type and *OsGRXS17* silenced plants was measured. Under water deficit conditions, all silenced plant lines showed lower water loss as compared to wild-type plants at 3 h (Fig. 5c). By 4 h, water loss rates of *OsGRXS17* RNAi-7, -8, and -13 were lower than wild-type and *OsGRXS17* RNAi-6 (Fig. 5c). The relative water content of all silenced lines was greater at 5 days of water deprivation in comparison to wild-type, although the water content of *OsGRXS17* RNAi-6 was intermediate to wild-type and the other silenced lines (Fig. 5d). At the same time, stomatal conductance was lower in *OsGRXS17* RNAi-7, -8, and -13 compared to wild-type and *OsGRXS17* RNAi-6 plants under both normal (Fig. 5e, 0 d) and water-deficit conditions (Fig. 5e). These results indicated that the enhanced drought tolerance of *OsGRXS17* silenced plants is caused by an increased ability of retaining water.

### Reduced *OsGRXS17* Expression Promotes Greater Stomatal Closure in Both the Presence and Absence of Exogenous ABA

**Figure 6:**
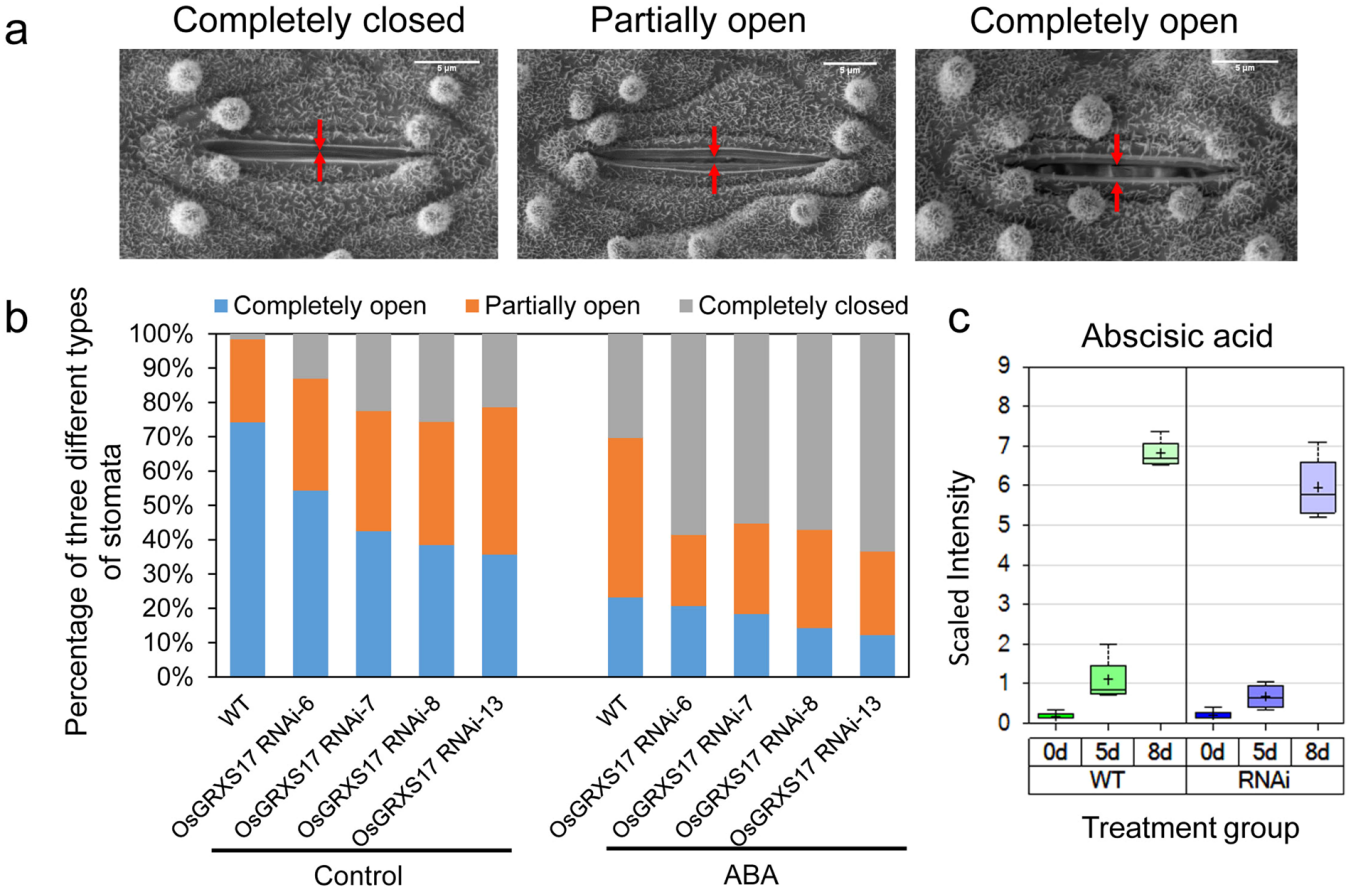
Comparison of stomatal opening status, and endogenous ABA level between wild-type and *OsGRXS17* silenced rice plants. (**a**) Scanning electron microscope images of three different statuses of rice stomata. Scale bars = 5 µm. (**b**) Percentage of three different types of stomata in four-week-old wild-type and *OsGRXS17* silenced rice plants under normal growth conditions or treated by 100 µM ABA (*n* = 62 stomata for wild-type, *n* = 58 stomata for *OsGRXS17* silenced-6, *n* = 55 stomata for *OsGRXS17* silenced-7, *n* = 65 stomata for *OsGRXS17* silenced-8 and *n* = 60 stomata for *OsGRXS17* silenced-13). (**c**) Endogenous ABA content of the wild-type and *OsGRXS17* silenced rice plants (*n* = 4). Error bars represent the means ± SD (*n* = 4).

Because *OsGRXS17* silenced plants showed reduced water loss rates and stomatal conductance, the effect of *OsGRXS17* expression on stomatal aperture was investigated. Stomatal aperture status, upon examination by scanning electron microscopy, was classified into three groups: closed, partially open, and open (Fig. 6a). The results indicated that reduced *OsGRXS17* expression led to a greater degree of stomata closure under normal growth conditions (Fig. 6b, left panel). In the extremes, wild-type plants had 2% closed stomata, 28% partially opened stomata, and 75% open stomata, while *OsGRXS17* RNAi-13 plants had 20% closed, 42% partially opened, and 38% open stomata (Fig. 6b, Control). Stomatal response to the application of ABA was then evaluated. All of the silenced lines showed greater stomatal closure compared to wild-type plants in the presence of ABA (Fig. 6b, ABA). No differences in stomatal density were found between *OsGRXS17* silenced lines and wild-type plants (Fig. S6). The results indicated that ABA treatment resulted in a higher percentage of closed stomata in *OsGRXS17* silenced rice plants in comparison to wild-type plants. To determine if the ABA hypersensitivity of the silenced lines was caused by over-accumulation of endogenous ABA, the levels of ABA were measured by metabolite profiling. ABA content and accumulation patterns were similar between the wild-type and silenced plants over the entire period of drought stress treatment (Fig. 6c), indicating that the increased stomatal closure in *OsGRXS17* silenced rice plants was not the result of ABA over-accumulation.

### Reduced Expression of *OsGRXS17* is Associated with the H_2_O_2_ Accumulation in Guard Cells

**Figure 7:**
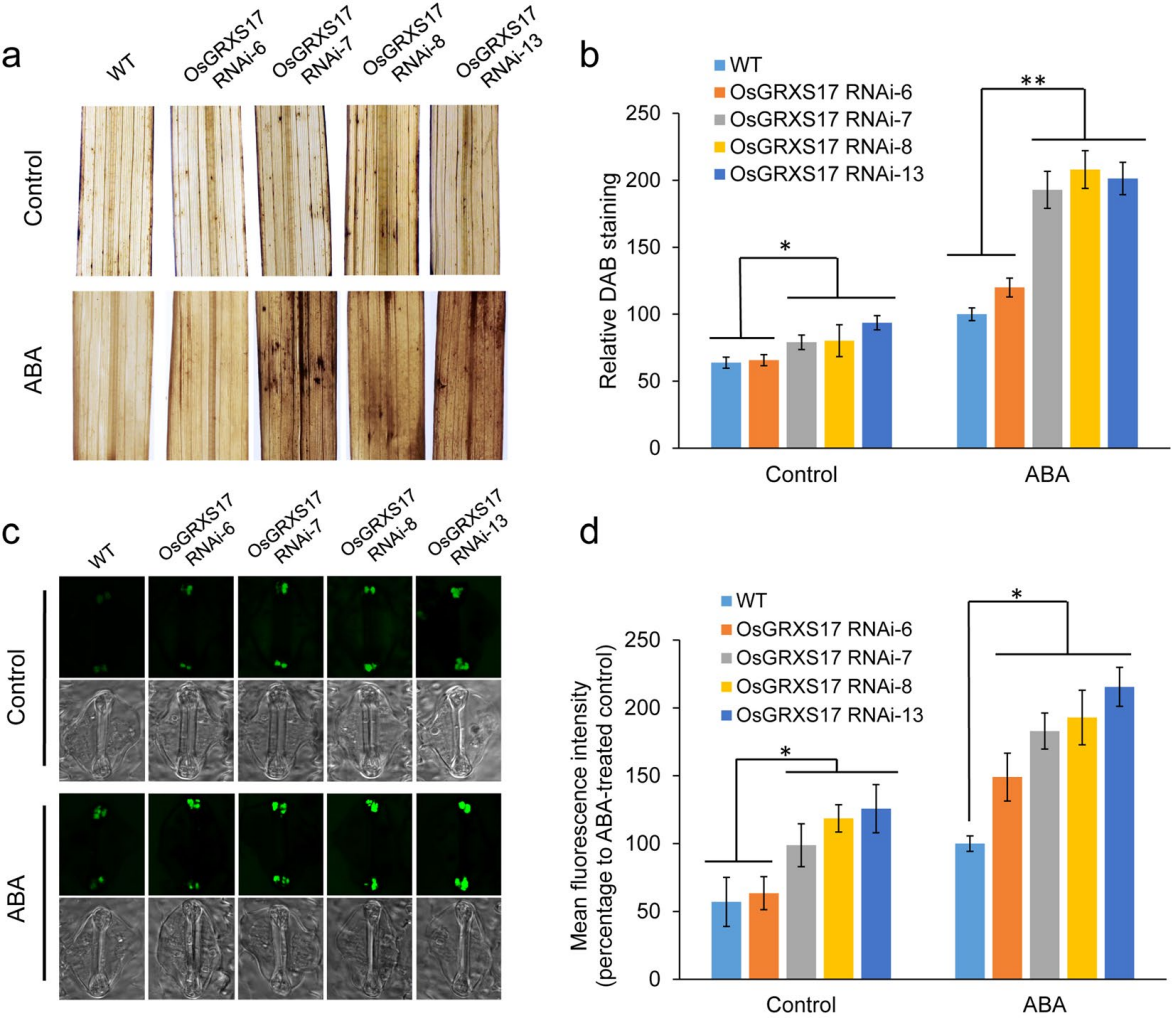
Effect of silenced expression of *OsGRXS17* on H_2_O_2_ accumulation. (**a**) DAB staining of the middle section of the first fully expanded leaf of four-week-old wild-type and *OsGRXS17* silenced rice plants. (**b**) Quantitative analysis of DAB staining. The relative intensity of DAB staining leaves was analyzed after being transformed to 256 gray scale images. Data are expressed as relative values based on wild-type plants treated by 100 µM ABA as reference sample set as 100. Error bars represent the means ± SD (*n* = 3 repeats, 6 plants in each repeat). Asterisks (*, **) represent statistically significant differences between wild-type and *OsGRXS17* silenced lines (Student’s *t-test*, *P < 0.05, **P < 0.01). (**c**) H_2_DCFDA staining of the guard cell of four-week-old wild-type and *OsGRXS17* silenced rice plants. (**d**) Quantitative analysis of H_2_DCFDA staining. Data are expressed as relative values based on wild-type plants treated by 100 µM ABA as reference sample set as 100. Error bars represent the means ± SD (*n* = 3 repeats, 6 plants in each repeat). Asterisks (*) represent statistically significant differences between wild-type and *OsGRXS17* silenced lines (Student’s *t-test*, *P < 0.05).

ABA-induced accumulation of H_2_O_2_, which is synthesized in guard cells, is essential for stomata closure by activating plasma membrane Ca^2+^ channels^5,37,38^. H_2_O_2_ accumulation was measured by staining the leaves of wild-type and *OsGRXS17* silenced plants using 3,3′-diaminobenzidine (DAB) and image analysis. In the absence of ABA, the leaves of *OsGRXS17* RNAi-7, -8 and -13 plants displayed slightly higher H_2_O_2_ levels on the basis of DAB staining than those of *OsGRXS17* RNAi-6 and wild-type plants (Fig. 7a, Control). In the presence of 100 µmol/L ABA, H_2_O_2_ accumulation was visibly increased in both wild-type and silenced leaves (Fig. 7a, ABA). However, the *OsGRXS17* RNAi-7, -8 and -13 plants displayed much stronger staining in comparison to leaves of *OsGRXS17* RNAi-6 and wild-type plants (Fig. 7a, ABA). Quantitative image analysis revealed higher H_2_O_2_ accumulation in *OsGRXS17* RNAi-7, -8 and -13 compared to that of *OsGRXS17* RNAi-6 and wild-type plants in both the absence of exogenous ABA (Fig. 7b, control) and presence of exogenous ABA (Fig. 7b, ABA).

H_2_O_2_ accumulation was also monitored in guard cells using the fluorescent dye, 2′,7′- dichlorodihydrofluorescein diacetate (H_2_DCFDA). The results showed that *OsGRXS17* RNAi-7, -8 and -13 lines had stronger fluorescence signal in the guard cell as compared to *OsGRXS17* RNAi-6 and wild-type plants in the absence of ABA addition, indicating that more accumulation of H_2_O_2_ in the guard cell of *OsGRXS17* RNAi-7, -8 and -13 (Fig. 7c, Control). In the presence of ABA addition, the fluorescence signal in the guard cells was enhanced in both wild-type and *OsGRXS17* silenced rice plants, with, again, *OsGRXS17* RNAi-7, -8 and -13 having stronger fluorescence signals in the guard cells as compared to *OsGRXS17* RNAi-6 and wild-type plants, indicating that higher accumulation of H_2_O_2_ in the guard cells of *OsGRXS17* RNAi-7, -8 and -13 lines than that of *OsGRXS17* RNAi-6 and wild-type plants under ABA treatment (Fig. 7c, ABA). The cytoplasm and nucleus of guard cells are dumbbell shaped and the two terminal masses are connected through a very thin central canal^39^. It is worth noting that the two terminals of rice guard cell have a fluorescence signal that is more easily detected while the central region is less noticeable due to the thickness of the guard cell wall in the central region, which is consistent with previous studies^40,41^. Quantitative analysis of fluorescence intensity also showed that H_2_O_2_ accumulation in the guard cell was higher in *OsGRXS17* RNAi-7, -8 and -13 compared with that of *OsGRXS17* RNAi-6 and wild-type plants with or without ABA treatment (Fig. 7d). A negative correlation between the *OsGRXS17* expression level (Fig. 4b) and the H_2_O_2_ accumulation in guard cells indicates that the increased ABA-induced stomatal closure in the *OsGRXS17* silenced lines was associated with higher H_2_O_2_ accumulation.

### Expression of ABA-Responsive Genes Is Elevated in *OsGRXS17* Silenced Plants

**Figure 8:**
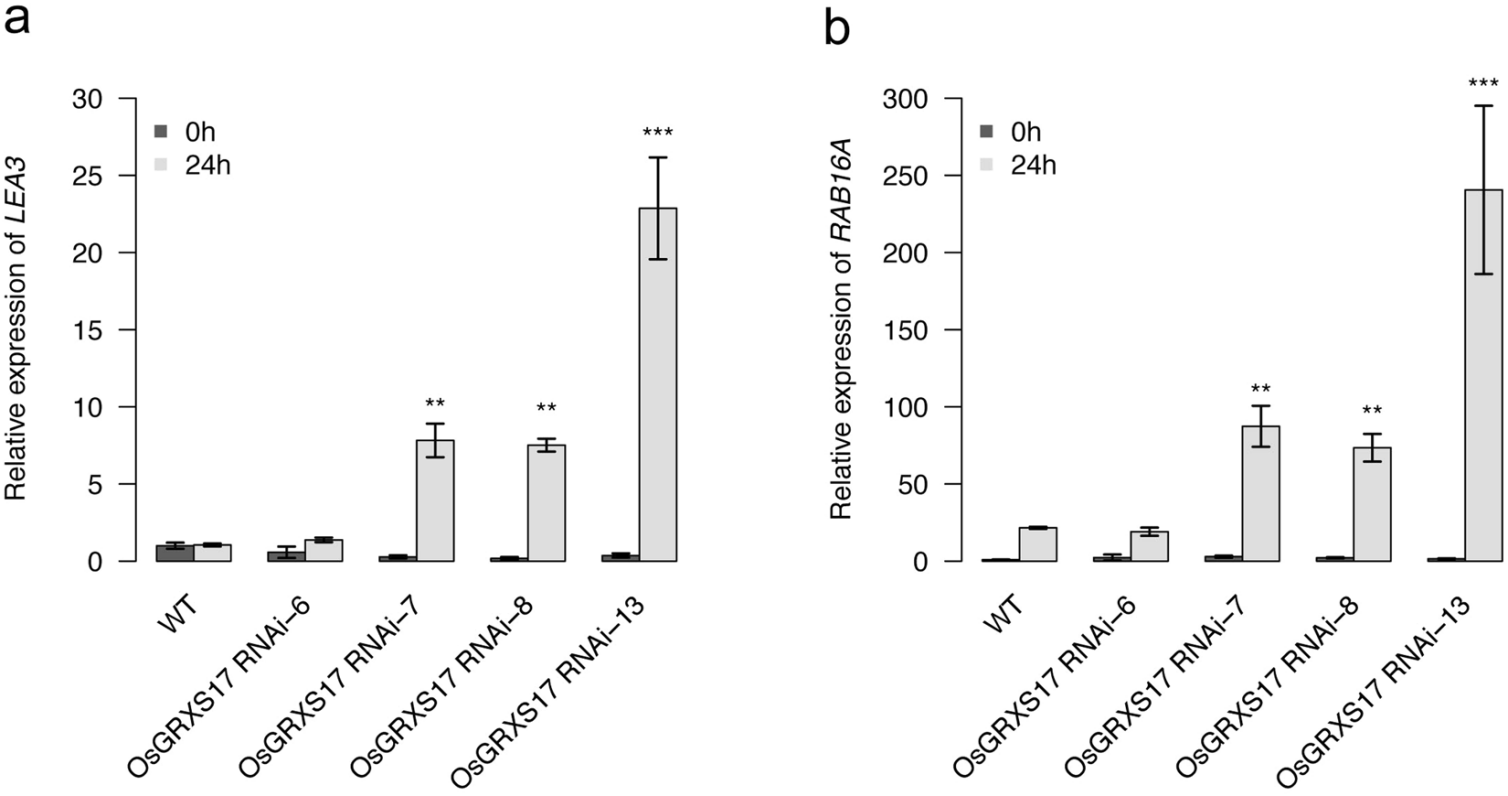
Expression analysis of ABA-responsive genes in wild-type and *OsGRXS17* silenced rice plants. Relative expression of *LEA3* (**a**) and *RAB16A* (**b**) in four-week-old wild-type and *OsGRXS17* silenced rice plants were detected by qRT-PCR after being treated by 100 µM ABA for 0 and 24 h, respectively. Data are expressed as relative values based on wild-type plants grown under control condition (0 h) as reference sample set to 1.0. Error bars represent the means ± SD (*n* = 3). Asterisks (**, ***) represent statistically significant differences between wild-type and *OsGRXS17* silenced lines (Student’s *t-test*, **P < 0.01, ***P < 0.001).

To examine if the ABA hypersensitivity and increased stomatal closure of the silenced lines associated with, at least in part, changes in ABA-responsive gene expression, the expression levels and patterns of four ABA-dependent drought-responsive genes (*RAB16A*, *LEA3*, *LIP9*, and *SalT*) and two ABA-independent genes (*DREB1A* and *DREB1E*)^42^ were analyzed in ABA-treated leaf samples by qRT-PCR. Inconsistent expression levels and patterns were detected for *LIP9*, *SalT*, *DREB1A* and *DREB1E* in both wild-type and *OsGRXS17* silenced rice plants. Expression levels of two ABA-responsive genes *LEA3* and *RAB16A* [belongs to LEA (late embryogenesis abundant) protein] were markedly increased in the *OsGRXS17* silenced RNAi-7, -8 and -13 rice plants in comparison to *OsGRXS17* RNAi-6 and wild-type plants within 24 h of ABA treatment, exhibiting 10- to 40-fold (*LEA3*) and 50- to 200-fold (*RAB16A*) elevated expression (Figs 8 and S7). To evaluate if drought stress responses and tolerance in the silenced lines is associated with altered regulation of the six selected ABA-dependent and/or ABA-independent drought-responsive genes, the expression levels and patterns of the six genes were also analyzed in drought stress treated-leaf samples. All six genes were highly upregulated after 24 h of drought stress treatment regardless of genotype (Fig. S8). These results suggest that repression of *OsGRXS17* selectively affects some, but not all ABA-responsive genes.

## Discussion

Previous studies have shown that the monothiol CGFS-type glutaredoxin GRXS17 plays important roles in plant abiotic stress adaptation, and that ectopic over-expression of *GRXS17* in plants enables enhanced heat and chilling stress tolerance^33–36,43^. Based on these reports, we hypothesized that reduction in *GRXS17* expression would result in plants that are more sensitive to abiotic stresses, including drought, compared to wild-type controls. Contrary to this hypothesis, here, suppression of *OsGRXS17* expression resulted in enhanced tolerance to the drought stress conditions in rice. Previous work had also shown that ROS, H_2_O_2_ in particular, are important signaling molecules in the regulation of stomatal aperture^44^. *OsGRXS17* silenced rice plants were found to have higher steady state H_2_O_2_ concentrations in guard cells in the absence of drought stress or exogenous ABA treatment. The increase in H_2_O_2_ accumulation is consistent with the observed increase in stomatal closure and increased sensitivity to the endogenous ABA compared to wild-type controls. Earlier studies have shown that antioxidant enzymes function in the stomatal closure via modulating ROS levels in guard cells. For example, a rice *dst* (drought and salt tolerance) mutant, lacking a zinc finger transcription factor, showed drought tolerance compared to wild-type plants. Further analyses indicated that DST bound directly to the promoter of genes encoding antioxidant enzymes that were negative regulators of H_2_O_2_ homeostasis^40^. Tobacco plants engineered to express higher level of *dehydroascorbate reductase* (*DHAR*) had an increase in ascorbic acid redox state, reduction in guard cell H_2_O_2_ concentrations, a reduction in stomatal closure, and an increase in susceptibility to drought while tobacco plants engineered to suppress expression of *DHAR* had a decrease in ascorbic acid redox state, an increase in guard cell H_2_O_2_ level, and increase in drought tolerance^45^. In addition, the H_2_O_2_-induced stomatal closure could be reversed by exogenous ascorbic acid treatment in a concentration-dependent manner^5^. Consistent with these reported studies, we found that guard cell H_2_O_2_ concentrations inversely correlated with *OsGRXS17* expression. The lower the expression of *OsGRXS17*, the more H_2_O_2_ accumulated in guard cells, indicating that OsGRXS17 plays an important role in modulating H_2_O_2_ homeostasis in guard cells.

Another factor modulating cellular H_2_O_2_ concentrations is ABA. ABA has been shown to induce H_2_O_2_ production in guard cells via the activation of plasma membrane NADPH oxidases^5,38,46^. H_2_O_2_ then acts as an essential second messenger in the ABA signaling pathway, mediating stomatal closure in response to drought stress^37,38,47,48^. In *Vicia faba*, ABA-induced stomatal closure was abolished by the presence of catalase (CAT), an H_2_O_2_ scavenger, and diphenylene iodonium (DPI), an inhibitor of NADPH oxidases, which remove H_2_O_2_ and reduce the production of H_2_O_2_, respectively^5^. Similarly, DPI has been shown to inhibit ABA-induced stomatal closure in *Arabidopsis* ^38^. In this study, *OsGRXS17* silenced rice plants had increased H_2_O_2_ in guard cells even without exogenous ABA treatment, possibly due to 1) the loss of a functional role of *OsGRXS17* as a ROS scavenger or 2) the hypersensitivity of *OsGRXS17* silenced lines to endogenous ABA. Further, we did find that *OsGRXS17* silenced rice plants accumulated more H_2_O_2_ after ABA treatment compared to wild-type rice plants. Since H_2_O_2_ plays an important role in mediating ABA-induced stomatal closure, it is likely that enhanced H_2_O_2_ accumulation is associated with increased stomatal closure in *OsGRXS17* silenced rice plants in the presence of ABA. However, we cannot still exclude the possibility that another parallel pathway may involve in stomatal closure via H_2_O_2_ production.

Application of exogenous ABA was also found to upregulate two ABA-dependent drought stress-responsive genes, *LEA3* and *RAB16A*, in the *OsGRXS17* silenced RNAi-7, -8 and -13 rice plants in comparison to *OsGRXS17* RNAi-6 and wild-type plants. These two genes; however, did not show different expression levels and patterns between wild-type and *OsGRXS17* silenced rice plants under drought stress treatment (Fig. S8). LEA proteins are a family of highly hydrophilic proteins that accumulate in mature seeds and vegetative tissues under dehydrated conditions and play an important role in protection of proteins and membrane stabilization. Overexpression of *OsLEA3* has been shown to enhance drought tolerance in rice^49^. Thus, the reduction of *OsGRXS17* expression may enhance drought tolerance by mediating ABA-regulated but not drought-regulated mRNA accumulation of *LEA* genes. Expression of other ABA-responsive and drought-responsive genes were not affected by *OsGRXS17* silencing under drought stress and exogenous ABA treatments, suggesting that OsGRXS17 might be involved in one portion of the ABA signaling pathways. OsGRXS17 could regulate ABA signaling through a post-translational modification mechanism. Measurements of endogenous ABA content revealed no significant difference between *OsGRXS17* silenced and the wild-type rice plants under drought stress, suggesting that OsGRXS17 might not be involved in the ABA biosynthesis pathways. Taken together, these results suggested that OsGRXS17 may play dual roles in a parallel way. It regulates H_2_O_2_ homeostasis, acting as a ROS scavenger, and involves in H_2_O_2_-mediated stomata closure. It may also participates in downstream signaling pathways of ABA by regulating the gene expression of *LEA3* and *RAB16A*.

As plants lose over 95% of water through transpiration, engineering of stomata activity is an important approach to enhance drought tolerance in plants^50^. To further characterize the drought tolerance of silenced *OsGRXS17* rice plants, we measured the water maintaining capacity and found the transgenic rice plants indeed showed reduced water loss rate, higher relative water content and reduced stomatal conductance compared to wild-type plants under drought stress, suggesting that increased ROS accumulation in *OsGRXS17* silenced rice plants might play an important role in drought stress response through regulating the redox-dependent signaling pathway. Although the *OsGRXS17* silenced plants exhibit reducing stomatal opening, no effect on rice grain yield was found (Fig. S2). Thus under both normal and drought stress conditions, CO_2_ influx is assumed to be sufficient to support normal photosynthesis and plant growth.

## Methods

### Yeast Assays

The full-length cDNA of *AtGRXS17* and *OsGRXS17* was subcloned into yeast expression vector piUGpd. Yeast *grx3grx4* double mutant (*MAT*a *ura3–52 leu2*Δ*1 his3*Δ*200 grx3::kanMX4 grx4::kanMX4*) were provided by Dr. Enrique Herrero (Universitat de Lleida, Lleida, Spain). Yeast growth assays were performed as previously described^29^.

### OsGRXS17 complementation assays in *atgrxs17* mutants

To understand the effect of OsGRXS17 on plant growth in *atgrxs17* mutants, an *Arabidopsis* expression cassette was developed. Full-length *OsGRXS17* was inserted into the entry vector (pENTR/D-TOPO vector, Invitrogen, Carlsbad, CA). The *35S*::*GFP-OsGRXS17* construct was made by LR clonase reaction between the entry vector and pB7WGF2 destination vector^51^ and was introduced into *A. tumefaciens* strain LBA4404 using the freeze-thaw method^52^. The *atgrxs17* mutant plants were transformed using the floral dip method^53^. Plants were screened by spraying a 1% BASTA solution on cotyledons. Seeds of wild-type, *atgrxs17* mutant and T2 transgenic *atgrxs17* mutants expressing *OsGRXS17* were placed on half-strength Murashige and Skoog (MS) media supplemented with 0.5% sucrose^54^. Seeds were allowed to germinate for three days at control temperatures followed by control (22 °C) or heat stress (28 °C) conditions for ten days supplemented with 150 µmol/m^2^/s light intensity.

### Subcellular localization of OsGRXS17 in plant cells

To study the subcellular localization of OsGRXS17 in plant cells, an *Agrobacterium*-mediated transient expression assay was conducted in tobacco leaves (N. *tabacum*) and polyethylene glycol (PEG)-mediated protoplast transformation was performed in protoplasts derived from rice leaf sheaths as described previously^55,56^. Full length *OsGRXS17* was inserted into the pENTR/D-TOPO cloning vector. The GFP-OsGRXS17 construct was produced by an LR clonase reaction between the entry vector and pB7WGF2^51^. As a control, a modified green fluorescent protein construct (Free GFP construct) was made by the Cre-loxP recombination system^57^. To function as a nuclear marker, pSK001 was generated by inserting a 1.9 kb *Sac*I-*Hind*III fragment from pBV579 (containing 35S::mCherry::NLS::Tnos) into the unique *Sac*I and *Hind*III sites of pCAMBIA1300. These three constructs were introduced into A. *tumefaciens* LBA4404 for transient expression in tobacco leaves. *A*. *tumefaciens* cells were cultivated overnight, and 5 mL of the culture was pelleted and re-suspended with infiltration medium (250 mg D-glucose, 5 mL MES stock solution, 5 mL Na_3_PO_4_•12H_2_O stock solution, 5 mL 1 M acetosyringone stock solution; made up to 50 mL final volume with ddH_2_O.) to an optical density of 0.1. *A*. *tumefaciens* cells were then infiltrated into tobacco leaves, and the infiltrated tobacco plants were kept under constant light for 1.5–2 days. The fluorescence signals were detected after 1.5–2 days of inoculation.

For PEG-mediated protoplast transformation, protoplasts derived from rice sheath tissues were used^56^. Rice seedlings were grown in a growth chamber with a 16 h light (28 °C)/8 h dark (22 °C) photoperiod. Sheath tissues from fifty 2-week-old seedlings were sliced into 1-mm strips with a razor blade and placed into a flask with the digestion solution (0.15 M sorbitol, 0.25 M sucrose, 35 mM CaCl_2_, 20 mM KCl, 1.5% Cellulase R10 (From *Trichoderma viride*, 7.5 U/mg), 0.75% Macerozyme (R10 Macerating enzyme from Rhizopus sp. RPI) and 10 mM MES-KOH (pH 5.7). Vacuum was applied to the samples in the dark for 30 mins and then incubated at room temperature for 2 hours with gentle shaking at 20–30 rpm. The digested tissues were filtered into a centrifuge tube using a 40 $\mu$m nylon mesh and then the mesh was rinsed with 20 mL W5 solution (0.1% glucose, 0.08% KCl, 0.9% NaCl, 1.84% CaCl_2_•2H_2_O, 2 mM MES-KOH, pH 5.7). After centrifuging at 100 g for 7 mins at room temperature, the protoplasts were collected from the interface between the digestion solution and W5. The protoplasts were washed with W5 solution twice, resuspended in 3 mL W5 solution and incubated on ice for 30 min. Then, W5 solution was removed and the protoplasts were resuspended in MMG solution (0.4 M mannitol, 15 mM MgCl_2_, 4 mM MES-KOH, pH 5.7). Ten micrograms of plasmid DNA and 100 $\mu$L protoplasts (adjusted to 10^6^ protoplasts/mL) were gently mixed with 130 $\mu$L PEG-calcium transfection solution (40% PEG4000, 0.2 M mannitol, 100 mM CaCl_2_). After incubation for 30 mins, the transfection mix was diluted with 500 $\mu$L W5 solution, centrifuged at 100 *g* for 2 mins, and then resuspended in 1 mL W5 solution. The fluorescence signals were detected after 18 hrs of incubation at room temperature.

Images were captured with a confocal laser scanning system (Leica, SP5 X, Leica Microsystems Inc., Buffalo Grove, IL, USA) and fluorescence microscope (Zeiss Axio-Plan, Carl Zeiss Microscopy, Thornwood, NY, USA). The fluorescence signals were detected at 510 nm (excitation at 488 nm) for GFP and at 610 nm (excitation at 587 nm) for mCherry.

### RNAi Plasmid Construction and Rice Transformation

Two isoforms of *OsGRXS17* (Os10g35720.1 and Os10g35720.2) were identified using Rice Functional Genomic Express Database (http://signal.salk.edu/cgi-bin/RiceGE). To knock down both isoforms of *OsGRXS17* gene, a 398-bp of the *OsGRXS17* gene at the 3′ ends of the coding sequence and 3′UTRs was amplified using a forward primer: 5′- CACCAGGGATCGTTGCGAAAGAAA-3′ and reverse primer: 5′- AGCAAACTCGATGGTCGACGGATG-3′ as the silenced-targeted region and subcloned into the pENTR/D-TOPO vector (Fig. S9). This 389 bp of knockdown target sequence covers 316 bp of coding sequence of the long isoform of *OsGRXS17* mRNA and 73 bp of 3′ UTR region of both isoforms (long and short isoforms) of *OsGRXS17* mRNA (Fig. S9), which was aligned against Rice Functional Genomic Express Database to avoid off-target problems. After verification by DNA sequencing, the Gateway™ cassette was introduced the pANDA vector^58^ by the LR recombination reaction. Recombination between pENTR vectors and destination vectors were performed according to the manufacturer’s instructions (Invitrogen, Carlsbad, CA).

The verified plasmid DNAs were introduced into *Agrobacterium tumefaciens* LBA 4404 using the freeze-thaw method^52^. Mature seed-derived callus from rice (*Oryza sativa L*. *Japonica*) cv. Nipponbare was used for *Agrobacterium*-mediated transformation^59^. After inoculating with *A. tumefaciens*, callus was transferred to regeneration medium for 4–10 weeks at 25 °C under a 16-h photoperiod. The regenerated shoots were transferred to rooting medium for four more weeks, then established in soil.

### Plant Materials and Growth Conditions

To analyze the expression pattern of *OsGRXS17* in response to stress and hormones, the 2-week-old wild-type seedlings were placed on dry filter paper or transferred to filter paper saturated with MS solution supplemented with 25% PEG, 100 μM ABA, 1 μM IAA or 200mM NaCl, respectively, as described previously^60^. T2-generation *OsGRXS17* silenced or wild-type rice seeds were surface-sterilized and geminated on MS medium with or without 40 mg/L hygromycin for 7 days, and the 7-d-old seedlings were transferred into small pots containing Baccto premium potting soil (Michigan Peat Company, Houston, Texas, USA) in growth chamber with a 16 h light (28 °C)/8 h dark (22 °C) photoperiod. The pots were kept in the flat-bottom trays containing water. For drought treatment, 3-week-old rice seedlings with three leaves appeared were exposed to drought stress treatments. Each pot was filled with the same amount of soil to provide similar soil humidity. The water was withheld from the trays for 11 days and then the stressed plants were re-watered to allow the wilted rice plants to recover. After 14 days of recovery, the survival rates (%) were calculated from the numbers of surviving plants per total tested plants. The plants were scored as viable if one or more new leaves appeared.

### Physiological Measurements

The measurement of relative water content (RWC) was performed as described previously^61^. Briefly, on the fifth day of withholding water treatment, the first fully expanded leaves were detached and the fresh weight (FW) was measured immediately. Then the leaves were completely immerged in the double distilled water overnight for rehydration. The turgid weight (TW) was measured after blotting the rehydrated leaves. Finally, the dry weight (DW) was measured after drying in an oven at 80 °C overnight. The RWC was calculated as follows: RWC = (FW−DW)/(TW−DW). The measurement of water loss rate was performed by placing the detached first fully expanded leaves on a laboratory bench and recording their fresh weight for 0, 1, 2, 3, 4, and 5h. It is expressed as percentage of initial fresh weight. The stomatal conductance (mmol m^−2^s^−1^) was measured in the first fully expanded leaf of 4-week-old wild-type and *OsGRXS17* silenced plants using a portable porometer (SC-1 Leaf porometer, Decagon Devices, Pullman, WA, USA) in the auto mode for 30 seconds. The stomatal conductance was followed after being treated under drought stress for 0, 1, 3, 5, 7, 9 and 11 days. To measure the stomata density, the first fully expanded leaves of 4-week-old wild-type and *OsGRXS17* silenced rice seedlings grown in the growth chamber were sampled. Imprints were made by coating the adaxial surface with clear nail polish. After a few minutes, the clear tape was used to peel off the nail polish and this was mounted onto microscope slides. The stomata density (number of stomata per unit area) was counted from three random areas on the leaf under a light microscope (Olympus CH30; Olympus, Tokyo, Japan).

### H_2_O_2_ Assays

H_2_O_2_ was visually detected in leaves of rice plants by *in situ* staining with 3,3′- Diaminobenzidine (DAB) as described previously with modification^43^. The first fully expanded leaves detached from 4-week-old wild-type and *OsGRXS17* silenced rice plants were vacuum-infiltrated in 0.01% Tween 20 for 5 mins and then treated with 100 µM ABA for 3 h. The sampled leaves were collected and incubated in DAB solution (1 mg/mL, pH 3.8; Sigma-Aldrich) for 24 h at room temperature in darkness. The leaves were then de-colorized in boiling ethanol (96%) for 10 min before photographing. For the root tips staining, the 7-day-old rice seedlings were incubated in DAB solution (0.1 mg/mL, pH 3.8; Sigma-Aldrich) for 2 h at room temperature in darkness. Then the root tips were rinsed by ddH_2_O for three times and mounted on microscope slides for photographing. Quantitative analyses of DAB staining were performed using image J analysis^43^.

The H_2_DCFDA staining assay was performed as previously to detect H_2_O_2_ production in the guard cell^40^. The first fully expanded leaves from 4-week-old wild-type and *OsGRXS17* silenced rice plants were vacuum-infiltrated in 0.01% Tween 20 for 5 min and then incubated in 2% (w/v) cellulose Onozuka RS (Sigma-Aldrich) at 37 °C for 5 h to facilitate peeling off the epidermal layers. The peeled epidermal strips were incubated in the loading buffer (10 mM Tris-HCl, 50 mM KCl at pH 7.2) and then transferred to the staining buffer (loading buffer containing 50 mM H_2_DCFDA) for 20 min. The stained epidermal strips were washed in the ddH_2_O for three times to remove the excess H_2_DCFDA and mounted on microscope slides to detect with a Zeiss LSM 780 laser-scanning confocal microscope (Carl Zeiss SAS, Jena, Germany) using following parameter settings: excitation 488 nm, emission 546 nm, 2% laser power percent, 16 Bit depth, image size 1024 × 1024 pixels, scanning speed 7. Fluorescence was analyzed using ImageJ software. Thirty to fifty guard cells were observed per treatment for three independent replicates.

### Scanning Electron Microscopy

The first fully expanded leaves detached from 4-week-old wild-type and *OsGRXS17* silenced rice plants treated with 100 µM ABA or drought for 3 h were used. The leaf segment (0.5 cm) were cut from the middle of the leaf, fixed by 2.5% glutaraldehyde in 0.1 M phosphate buffer at pH7.2. The samples were then rinsed 3 times in distilled water, dehydrated in ethanol series (30, 50, 70, 80, 95%), and rinsed 3 times in 100% ethanol. The samples were finally critical point dried using hexamethyldisilazane (HMDS)^62,63^. The dehydrated samples were then sputter-coated with gold and used for stomatal observation by using a Hitachi S-3500N scanning electron microscope (Hitachi, Tokyo, Japan). Thirty to fifty guard cells were observed per treatment for three independent replicates.

### RNA Extraction and qRT-PCR

Total RNA was isolated from leaves, stems, roots and panicles using the Qiagen Plant RNeasy kit (Qiagen, Valencia, CA) and on-column DNA digestion according to the manufacturer’s instructions. The cDNA was transcripted from 1 µg quantity of total RNA using iScript™ Select cDNA Synthesis Kit (Biorad, Hercules, CA). The qRT-PCR was carried out in a total volume of 10 µL containing 4.2 µL the reverse-transcribed product, 0.4 µL 10 mM of each primer, and 5 µL SYBR Green PCR Master Mix (Bio-Rad). The PCR was performed with a Bio-Rad CFX-96 real time system (BioRad). Primer efficiencies were measured and relative expression level was calculated using the comparative Ct method. TFIIAγ5 was used as the internal control to normalize the samples. The primers used for qRT-PCR were listed in the supplemental Table 1.

### DNA Gel-blot Analysis

Rice genomic DNA was extracted from 4-week-old rice seedlings using CTAB method as described previously^64^. HindIII-digested genomic DNA (30 µg) was separated by electrophoresis on 0.8% (w/v) agarose gel, and blotted onto a nylon membrane (Zeta-probe GT membrane, Bio-Rad, Hercules, CA) according to the manufacturer’s instructions. The probe for the *hpt* gene was isolated from a pIPKB007 vector by PCR amplification. The membranes were pre-hybridized at 65 °C in 7% SDS and 0.25 M Na_2_HPO_4_ for 2 h and then hybridized overnight at 65 °C in the same solution containing the probe labeled with the enzyme horseradish peroxidase (ECL Direct™ Nucleic Acid Labeling and Detection System, Amersham Biosciences, Piscataway, NJ, USA) for 10–12 h at 42 °C. Membranes were washed twice for 40 min each with 20 mm Na_2_HPO_4_ and 5% SDS at 65 °C and then washed twice again for 30 min each with 20 mm Na_2_HPO_4_ and 1% SDS at 65 °C. Finally, the membrane was wrapped in Saran Wrap and exposed to X-ray film (Fuji Film Medical Systems, Stamford, CT) for 1–2 h.

### Measurement of ABA content by metabolite profiling analysis

First fully expanded leaves from 4-week-old wild type and *OsGRXS17* silenced rice plants were collected after 0 (0d), 5 (5d) and 8 (8d) days of drought stress treatment. Four biological replicates from each line were collected at each time point, lyophilized and kept at −80 °C until extraction. One hundred mg of lyophilized samples was submitted to Metabolon, Inc. (Durham, NC), for sample extraction and metabolite profiling analysis. The ABA content is provided by metabolite profiling analysis^62^. In brief, each sample was thawed on ice and extracted using an automated MicroLab STAR system in 400 µL of methanol containing recovery standards. A series of organic and aqueous extractions were performed to remove the protein fraction and also allow maximum recovery of small molecules. The extract was divided for two analyses: one for HPLC/MS analysis and the other for GC/MS analysis. Compounds were identified by comparison to library entries of purified standards or recurrent unknown entities.

### Data Availability

Sequence data from this article can be found in the Rice Functional Genomic Express Database and GeneBank/EMBL database under the following accession numbers: Os10g35720 (*OsGRXS17*), Os10g35720.1 (long isoform *OsGRXS17*), Os10g35720.2 (short isoform *OsGRXS17*), AK067982.1 (*OsGRXS17*), NM_001074376 (*RAB16A*), NM_001062730 (*LEA3*), XM_015755426 (*DREB1A*), XM_015779684.1 (*DREB1E*), XM_015766617.1 (*SalT*), XM_015771723.1 (*LIP9*), AY587109.1 (*AP59*), KM262835.1 (*AP37*), and NM_001060961 (*TFIIAγ5*).

## Electronic supplementary material

Supplementary Information
