# Supplementary material for: Silencing of OsGRXS17 in rice improves drought stress tolerance by modulating ROS accumulation and stomatal closure
Source: Sci Rep. 2017 Nov 21;7:15950. doi: 10.1038/s41598-017-16230-7 (PMC5698295; doi:10.1038/s41598-017-16230-7)
Supplement: Supplementary file 1 — Supplementary Information [file 41598_2017_16230_MOESM1_ESM.pdf]

Supplementary Information:
Silencing of *OsGRXS17* in rice improves drought stress tolerance by modulating ROS accumulation and promoting ABA-mediated stomatal closure

Ying Hu[1,5], Qingyu Wu[1,6], Zhao Peng[2,7], Stuart A. Sprague[1], Wei Wang[2], Jungeun Park[1], Eduard Akhunov[2], Krishna SV Jagadish[3], Paul A. Nakata[4], Ninghui Cheng[4], Kendal D. Hirschi[4], Frank F. White[2,7,*] and Sunghun Park[1,*]

[1]Department of Horticulture and Natural Resources, Kansas State University, Manhattan, KS 66506, USA
[2]Department of Plant Pathology, Kansas State University, Manhattan, KS 66506, USA
[3]Department of Agronomy, Kansas State University, Manhattan, KS 66506, USA
[4]United States Department of Agriculture/Agricultural Research Service, Children's Nutrition Research Center, Department of Pediatrics, Baylor College of Medicine, Houston, TX 77030, USA

Table S1 Primers used for qRT-PCR

Gene name	Accession #	Primer sequence
OsGRXS17	AK067982.1	Forward: TTCAGTACGGTTATATTGCCAGTT<br>Reverse: AAGCACCCTCTATTAATTGCGATA
RAB16A	NM_001074376	Forward: CCAGTTCCAGCCGATGAG<br>Reverse: TCCTCCCTCCCATTCCAT
LEA3	NM_001062730	Forward: ACCAAGGACTCTGCCATC<br>Reverse: GCTCTTCACCTGCTCACT
DREB1A	XM_015755426	Forward: GGCTGGGACCTGTACTAC<br>Reverse: CTAGTAGCTCCAGAGTGG
DREB1E	XM_015779684.1	Forward: CACAGGGAATGCTCATCG<br>Reverse: CAGTAGCTCCATAGATTGACC
SalT	XM_015766617.1	Forward: TGACAAGTGCTAATAATACATACG<br>Reverse: GTTCCAGACCTTCCAAAGA
LIP9	XM_015771723.1	Forward: GAAGACGCAACTGCTGTG<br>Reverse: CTGCTCACTTCCTCTGTAG
AP59	AY587109.1	Forward: CAACAAGCGGCCATATCC<br>Reverse: GAAGACGACGACGAGGAG
AP37	KM262835.1	Forward: GCAGCAATAGCACGGTAG<br>Reverse: AACAAATCCAAGTCCAGAGTC
TFIIAγ5	NM_001060961	Forward: GGGTTTGCCTGGTATTTGTTAG<br>Reverse: GTTGCTGCTGTGATATACTCTG

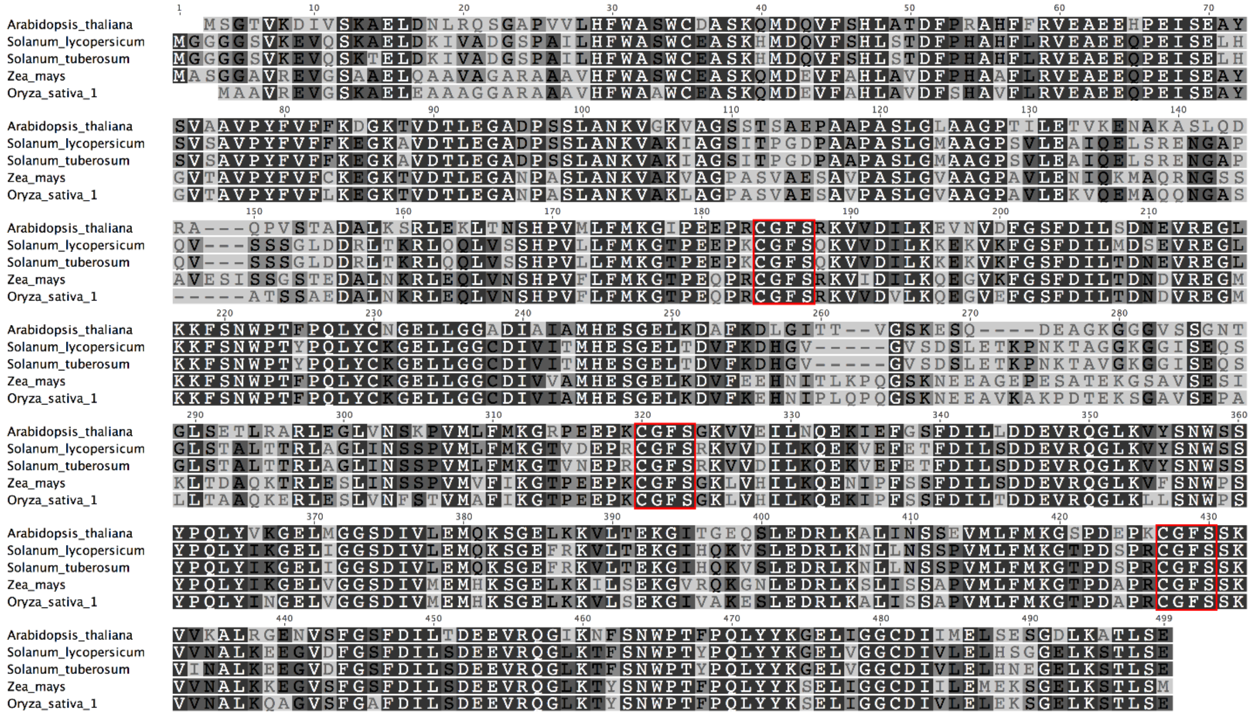

Fig. S1 Multiple amino acid sequence alignment of GRXS17 in *Arabidopsis*, tomato (*Solanum lycopersicum*), potato (*Solanum tuberosum*), maize (*Zea mays*) and rice (*Oryza sativa*, long isoform). Completely conserved residues are indicated by black boxes and residues conserved in the majority of sequences are indicated by gray boxes. The CGFS active domains are indicated by red border rectangles.

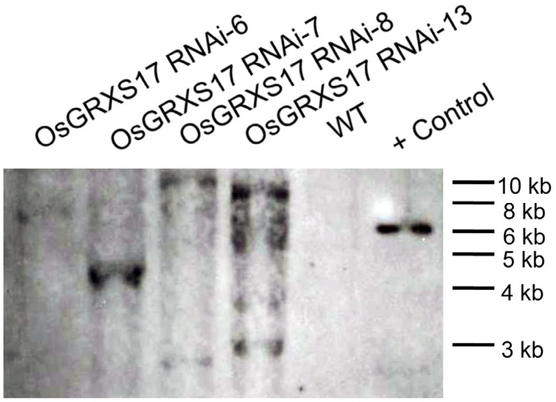

Fig. S2 Southern-blot analysis of T-DNA copy number of the *OsGRXS17* transgene. DNA probe of a *hygromycin phosphotransferase* gene was used for hybridization.

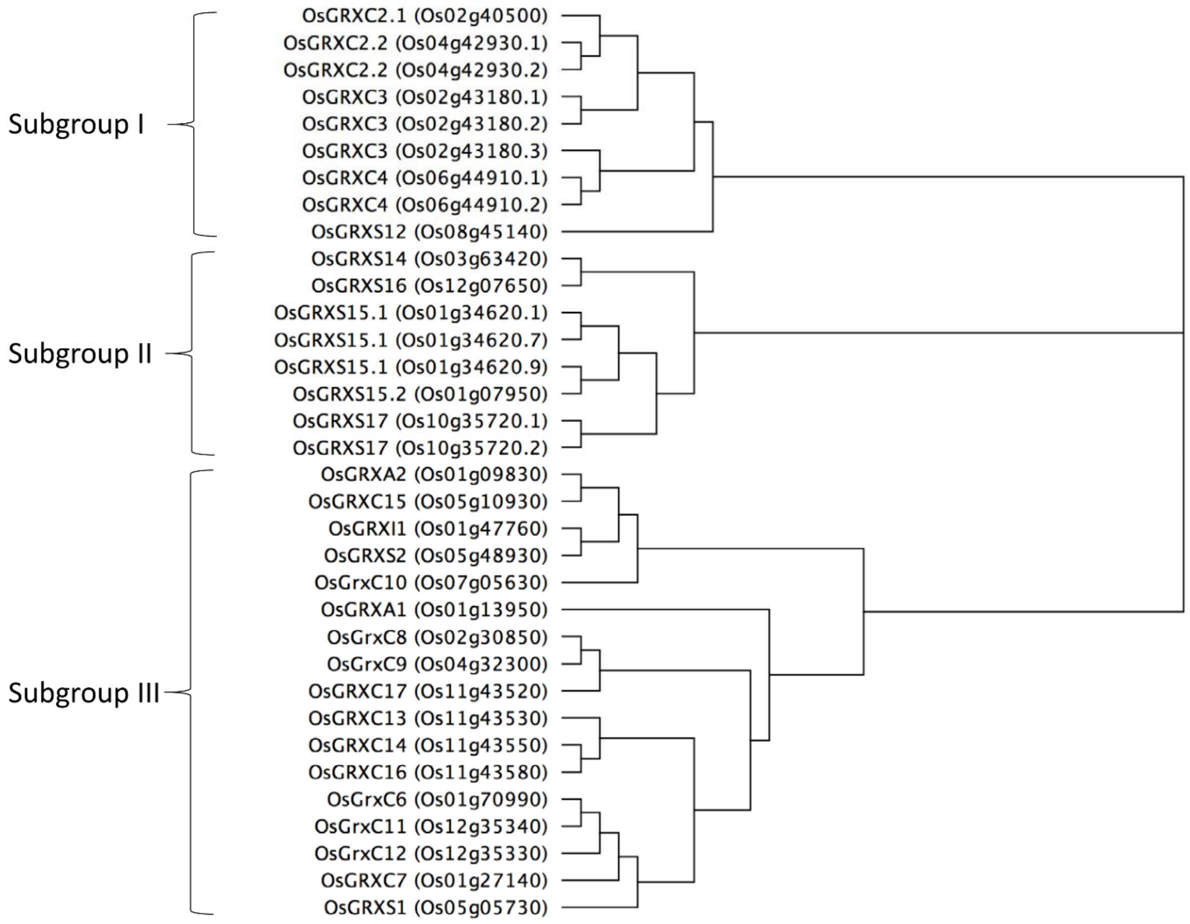

Fig. S3 Phylogenetic tree of rice glutaredoxin genes in subgroup I, II and III.

a

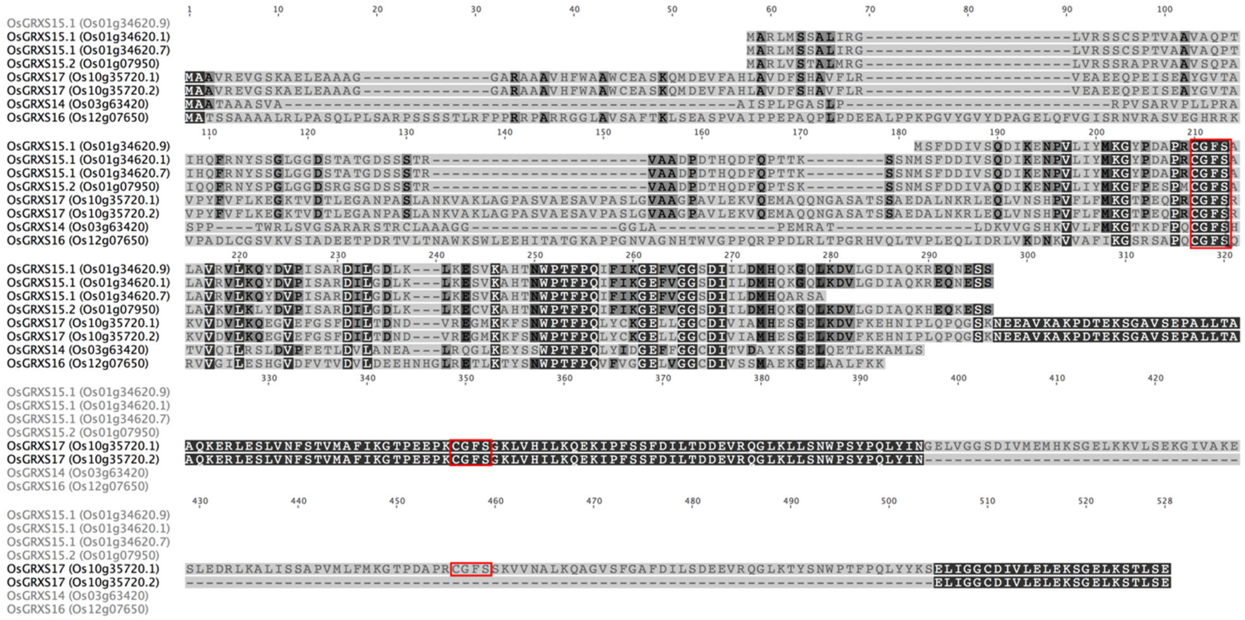

b

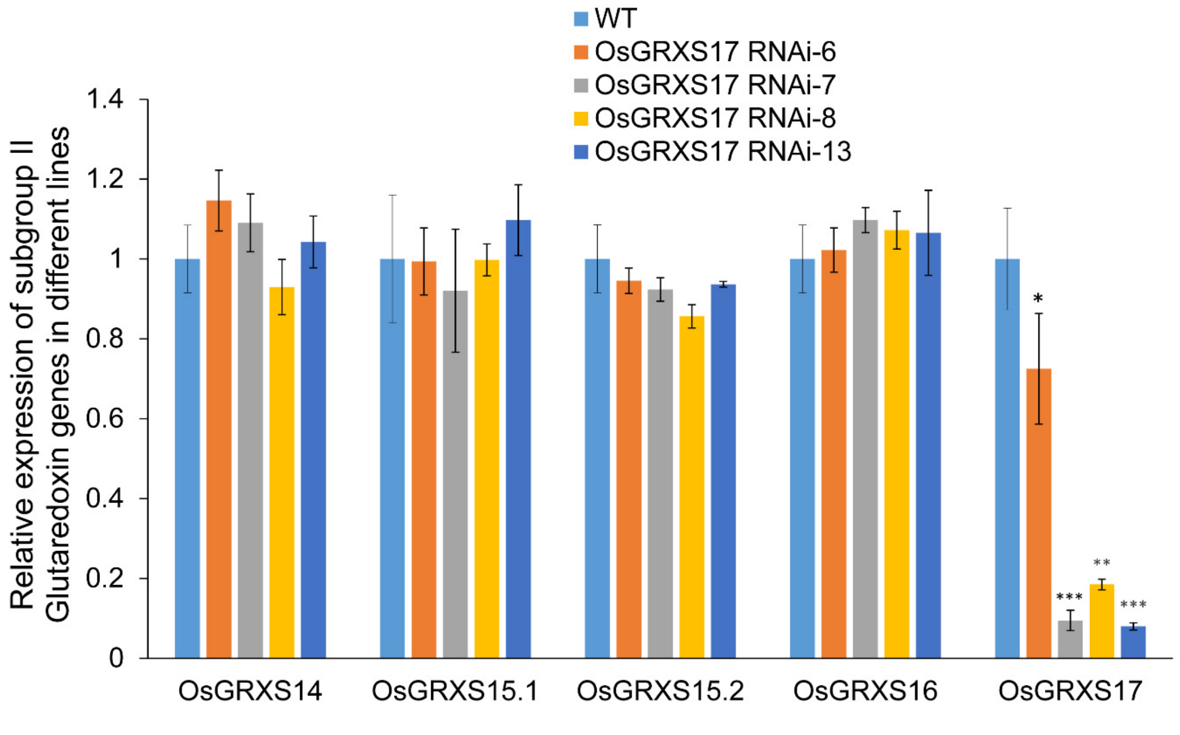

Fig. S4 (a) Multiple amino acid sequence alignment of rice glutaredoxin genes in subgroup II. Completely conserved residues are indicated by black boxes and residues conserved in the majority of sequences are indicated by gray boxes. The CGFS active domains are indicated by red border rectangles. (b) The relative expression levels of subgroup II glutaredoxin genes were measured by qRT-PCR in wild-type and four *OsGRXS17*-silenced rice plants.

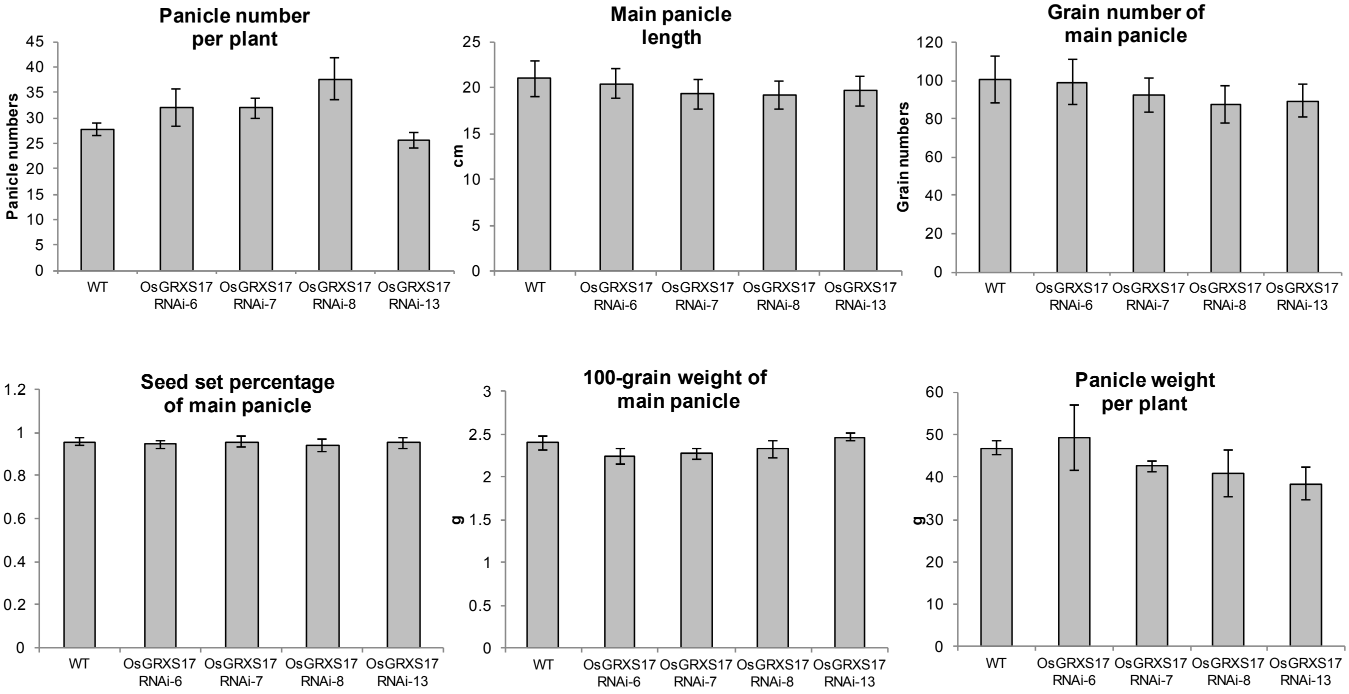

Fig. S5 Comparison of agronomic traits between wild-type and *OsGRXS17* silenced rice plants.

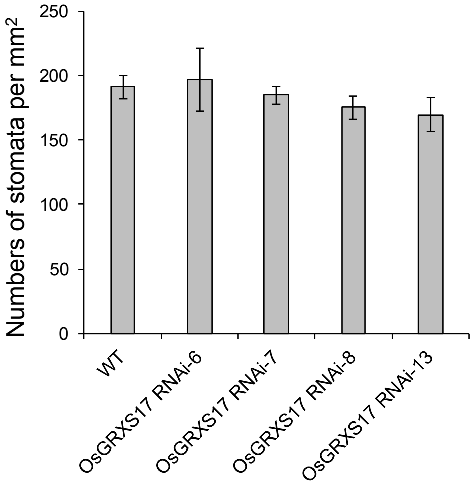

Fig. S6 Comparison of stomatal density between wild-type and *OsGRXS17* silenced rice plants.

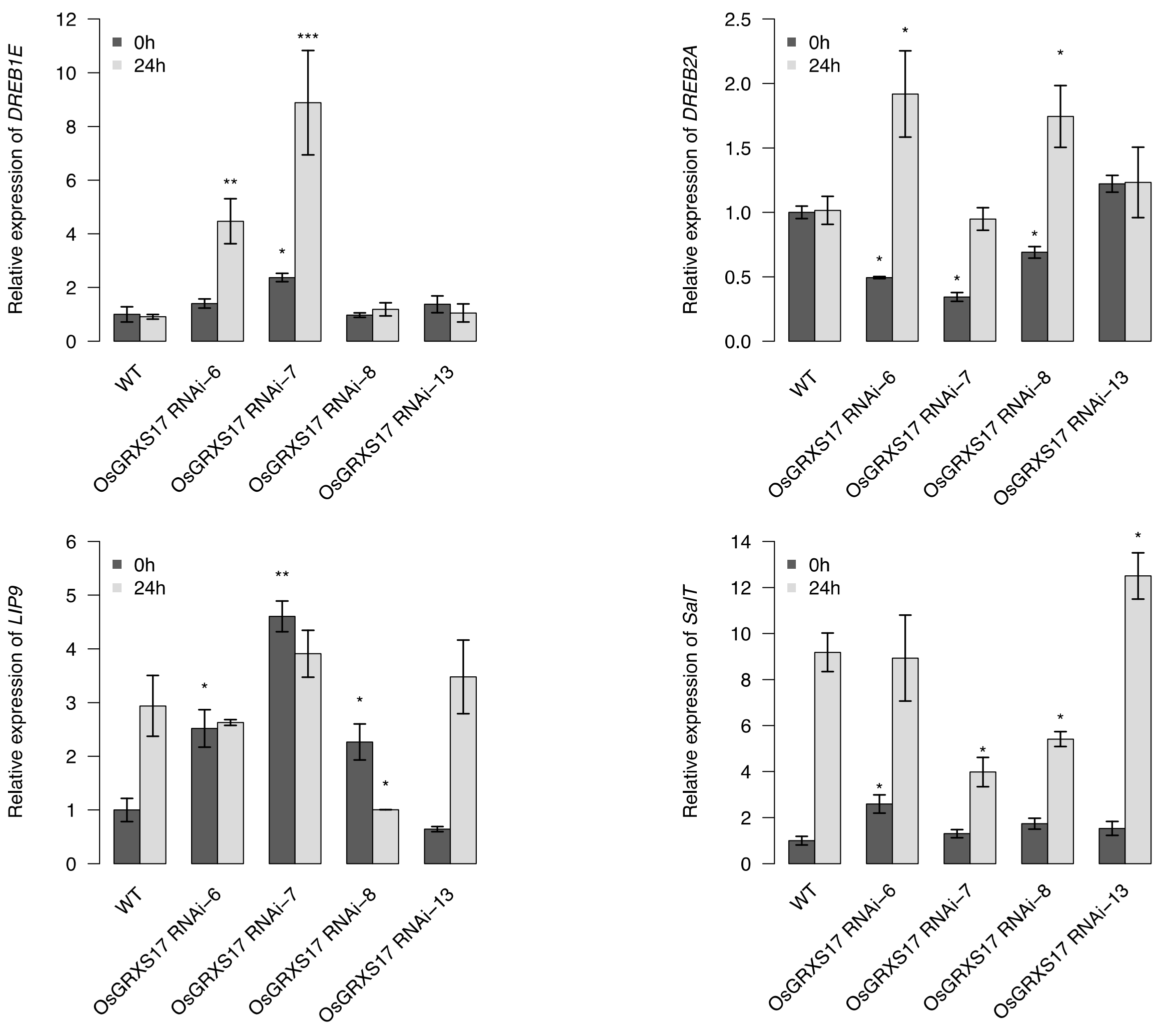

Fig. S7 Relative expression of four selected ABA-dependent and/or ABA-independent drought-responsive genes under ABA treatment.

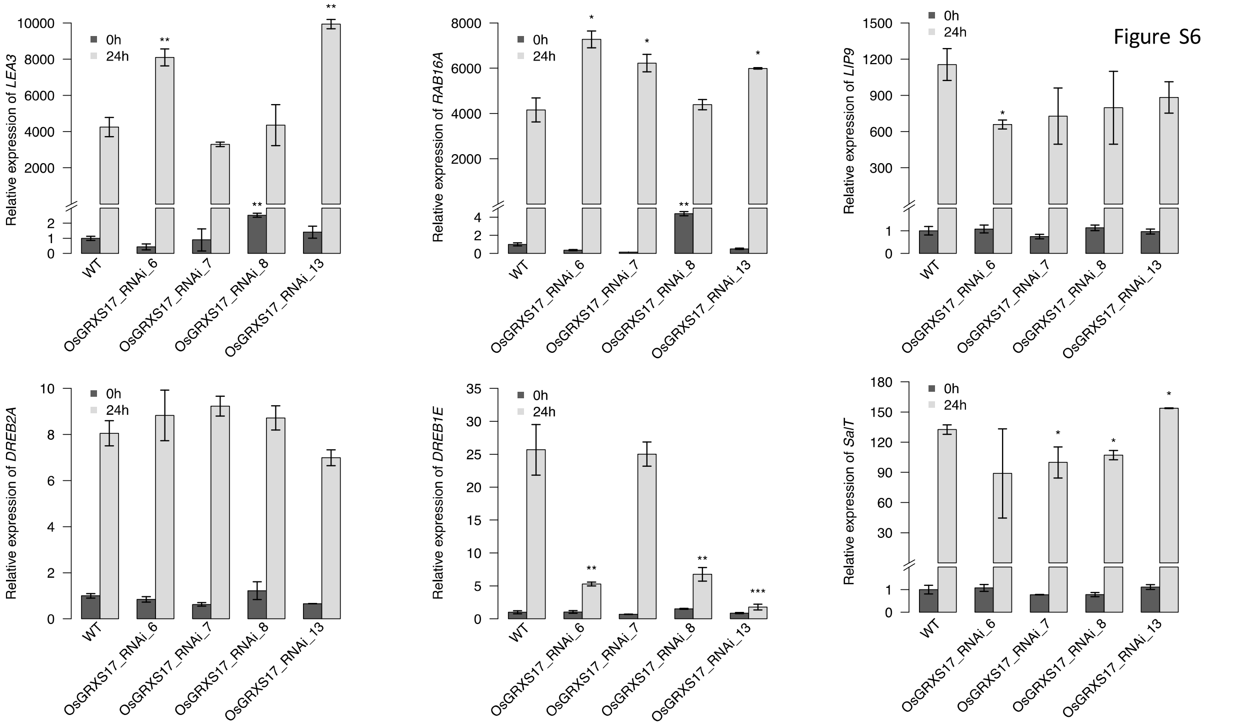

Fig. S8 Relative expression of six selected ABA-dependent and/or ABA-independent drought-responsive genes under drought treatment.

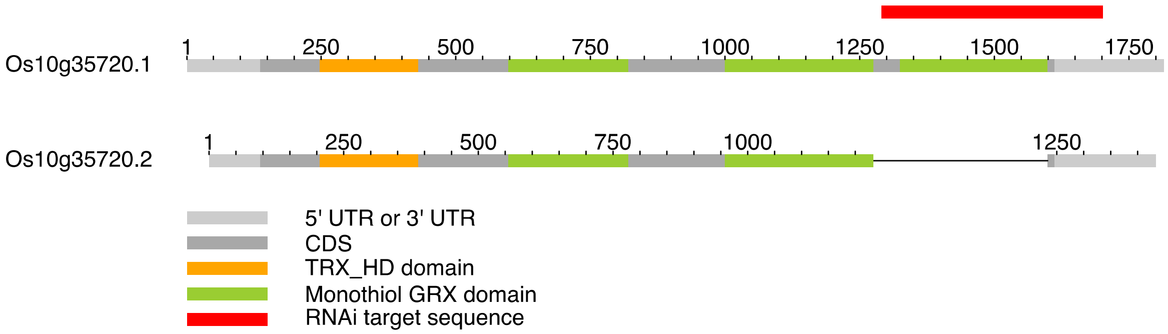

Fig. S9 Domain structure of two alternatively spliced *OsGRXS17* mRNAs. Red boxes indicate Thioredoxins_like homology domains; purple boxes indicate monothiol glutaredoxin domains; Orange boxes indicate RNAi target sequence.

(Full-length gels)

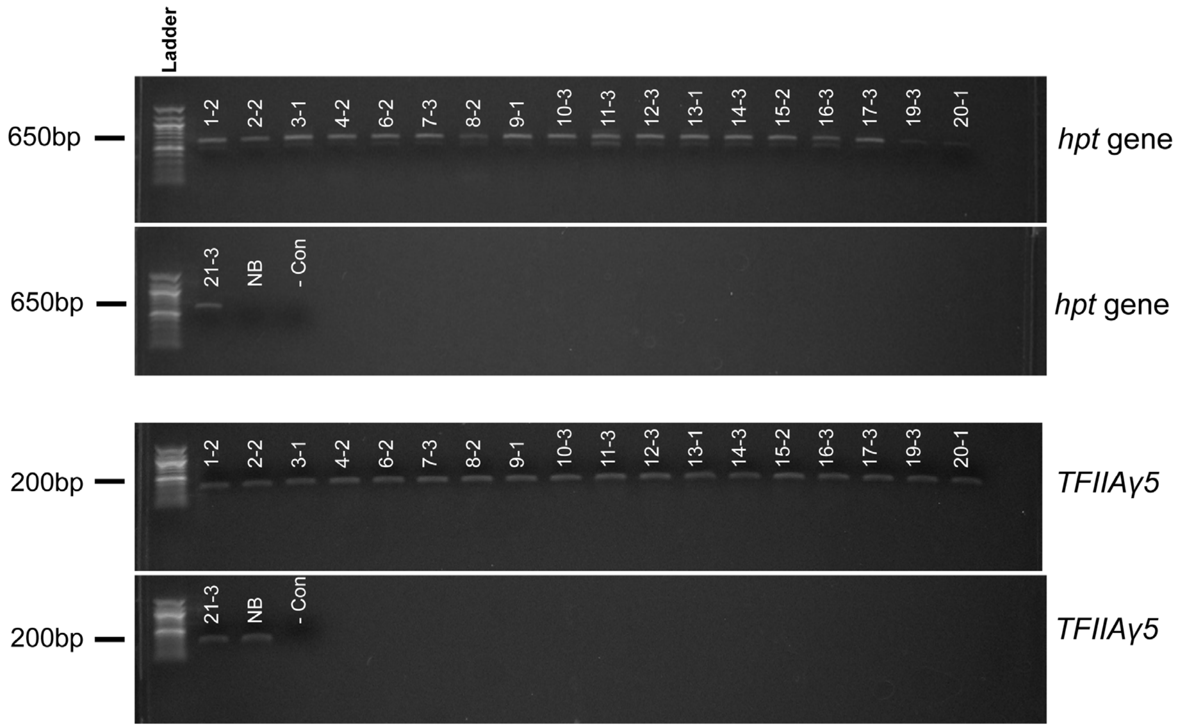

Fig. S10 Molecular characterization of *OsGRXS17*-silenced transgenic rice plants (progeny lines). The expression of a *hygromycin phosphotransferase* (*hpt*) gene was confirmed by RT-PCR in wild-type (NB) and nineteen *OsGRXS17*-silenced rice plants. The *TFIIAγ5* gene was included as control for uniform RT-PCR conditions (bottom).
